# Tracking microRNA Processing Signals by Degradome Sequencing Data Analysis

**DOI:** 10.3389/fgene.2018.00546

**Published:** 2018-11-14

**Authors:** Dongliang Yu, Min Xu, Hidetaka Ito, Weishan Shao, Xiaoxia Ma, Huizhong Wang, Yijun Meng

**Affiliations:** ^1^College of Life and Environmental Sciences, Hangzhou Normal University, Hangzhou, China; ^2^Faculty of Science, Hokkaido University, Sapporo, Japan

**Keywords:** microRNA annotation, degradome, tissue-/cell line-specific, “loop-to-base” processing, single-stranded cropping

## Abstract

Degradome sequencing (degradome-seq) was widely used for cleavage site mapping on the microRNA (miRNA) targets. Here, the application value of degradome-seq data in tracking the miRNA processing intermediates was reported. By adopting the parameter “signal/noise” ratio, prominent degradome signals on the miRNA precursors were extracted. For the 15 species analyzed, the processing of many miRNA precursors were supported by the degradome-seq data. We found that the supporting ratio of the “high-confidence” miRNAs annotated in miRBase was much higher than that of the “low-confidence.” For a specific species, the percentage of the miRNAs with degradome-supported processing signals was elevated by the increment of degradome sampling diversity. More interestingly, the tissue- or cell line-specific processing patterns of the miRNA precursors partially contributed to the accumulation patterns of the mature miRNAs. In this study, we also provided examples to show the value of the degradome-seq data in miRNA annotation. Based on the distribution of the processing signals, a renewed model was proposed that the stems of the miRNA precursors were diced through a “single-stranded cropping” mode, and “loop-to-base” processing was much more prevalent than previously thought. Together, our results revealed the remarkable capacity of degradome-seq in tracking miRNA processing signals.

## Introduction

Degradome sequencing (Addo-Quaye et al., 2008), also referred to as PARE (German et al., 2008) or GMUCT (Willmann et al., 2014), is an efficient strategy developed for transcriptome-wide detection of the uncapped 5′ ends of the polyadenylated RNAs. Degradome-seq was widely used for mapping the cleavage sites on the target transcripts of miRNAs or siRNAs, especially in plants (Addo-Quaye et al., 2008; German et al., 2008; Yu et al., 2017).

In plants, the miRNA precursors are processed by DCL1 through two-step cropping in the nucleus. However in animals, the two-step processing occurs in different subcellular locations. The first cropping by Drosha takes place in the nucleus, while the second cropping by Dicer occurred in the cytoplasm (Carthew and Sontheimer, 2009). It is well-known that most of the miRNA genes are transcribed by RNA polymerase II in both animals (Lee et al., 2004) and plants (Xie et al., 2005), resulting in the polyadenylated primary transcripts. In this regard, it was proposed that the processing intermediates of the miRNA precursors could be detected by degradome-seq (Meng et al., 2010).

In this study, the degradome-seq data of 15 species (11 plants and 4 animals), including *Arabidopsis thaliana* (Ath for short hereafter), *Brachypodium distachyon* (Bdi), *Glycine max* (Gma), *Medicago truncatula* (Mtr), *Oryza sativa* (Osa), *Physcomitrella patens* (Ppt), *Prunus persica* (Ppe), *Solanum lycopersicum* (Sly), *Solanum tuberosum* (Stu), *Vitis vinifera* (Vvi), *Zea mays* (Zma), *Caenorhabditis elegans* (Cel), *Drosophila melanogaster* (Dme), *Homo sapiens* (Hsa), *Mus musculus* (Mmu), was retrieved from the public databases to investigate their ability in tracking miRNA processing signals. As a result, the processing of a considerable portion of the analyzed miRNAs was found to be supported by degradome-seq data. Notably, for a specific species, the percentage of the miRNAs with degradome-supported processing signals (defined as “supporting ratio” hereafter) was elevated the increment of degradome sampling diversity to some extent. Besides, combined with sRNA-seq data analysis and secondary structure prediction, the degradome-seq data showed its great potential for the improvement of the current miRNA annotation accuracy. The analytical results of *Mus musculus* and *Homo sapiens* showed that the tissue- or cell line-specific accumulation pattern of the mature miRNAs could be partially reflected by the degradome-seq data, indicating that the miRNA processing pattern might be partially linked to the miRNA accumulation pattern. Finally, based on the distribution of the degradome-supported processing signals on the miRNA precursors, a renewed model was proposed that the double-stranded stem regions of the precursors were diced through a “single-stranded cropping” mode (i.e., cropping one strand at a time), and the “loop-to-base” processing might be much more prevalent than previously thought. Taken together, our results revealed the noteworthy potential of degradome-seq data in tracking miRNA processing signals, which might be also valuable for the study on miRNA annotation.

## Materials and Methods

### Data Sources and Bioinformatics Tools

All of the miRNA information (including sequences, genomic positions, and confidence annotations of the mature miRNAs and their precursors) was retrieved from miRBase (release 21^1^) (Kozomara and Griffiths-Jones, 2014). The genomes of the six model species were used to collect the 3′ 50-nt sequences downstream of the miRBase-registered miRNA precursors. Specifically, the genome sequences of *Arabidopsis thaliana, Caenorhabditis elegans, Drosophila melanogaster, Homo sapiens, Mus musculus* and *Oryza sativa* were retrieved from TAIR (The *Arabidopsis* Information Resource, release 10^2^) (Huala et al., 2001), Ensembl WBcel235^3^ (The C. elegans Sequencing Consortium, 1998), BDGP (Berkeley Drosophila Genome Project, release 5.0^4^) (Rubin, 1996), NCBI Human Genome Resources (GRCh38^5^) (Lander et al., 2001), NCBI mouse genome (GRCm38^6^) (Mouse Genome Sequencing et al., 2002), and RGAP (Rice Genome Annotation Project, release 7^7^) (Kawahara et al., 2013), respectively.

The degradome-seq datasets of 15 species were retrieved from GEO^8^ (Edgar et al., 2002), SRA^9^ (Leinonen et al., 2011), or Next-Gen Sequence Databases^10^ (Nakano et al., 2006). See Supplementary Table S1 for detail. The SPARE data of *Arabidopsis thaliana* was retrieved from SRA under the accession ID SRR835483.

The sRNA-seq datasets of *Arabidopsis thaliana* (accession ID: GSM707678), *Homo sapiens* (GSM494811 and GSM1666320), *Mus musculus* (GSM1666315 and GSM1666319), and *Zea mays* (GSM381716) were retrieved from GEO.

The Venn diagrams were drawn by using a online tool^11^. Secondary structure prediction of the miRNA precursors were performed by using RNAshapes (Steffen et al., 2006) with default parameter setting. Conserved sequence motif discovery was performed by using WebLogo 3 (Crooks et al., 2004).

### Pre-treatment of Degradome-Seq and sRNA-Seq Data

After removing the sequencing adapters and the low-quality reads containing “N,” the raw read count of each short sequence belonging to a specific sequencing dataset was normalized in RPM, thus enabling cross-dataset comparison. Specifically, the normalized read count of a short sequence was calculated through dividing the raw count of this sequence by the total raw counts of all short sequences within the dataset, and then multiplied by 10^6^.

### Searching for the Prominent Degradome Signals on the miRNA Precursors

The algorithm adopted to search for the prominent degradome signals on the miRNA precursors were reported in our previous study on the identification of cleavage signals on the miRNA targets (Shao et al., 2013). Specifically, for each degradome-seq dataset, only the perfectly mapped degradome signatures were retained, and the following parameters were defined. “Averaged read count of the potential slicing signals” (short for “signal”) is defined as the averaged read count (RPM) of the degradome signatures with their 5′ ends mapped to the potential slicing site. “Averaged read count of the surrounding signals” (short for “noise”) is defined as the averaged read count (RPM) of the degradome signatures mapped onto the miRNA precursor, except for those mapped to the potential slicing site. In default, the degradome signal will be considered to be prominent only when the “signal/noise” ratio ≥ 5. Besides, the most abundant degradome signature mapped to the potential slicing site should rank among the top 12 most-abundant signatures mapped onto the miRNA precursors. The “top 12” parameter was adopted from the pioneer work on the identification of miRNA cleavage sites based on degradome-seq data (German et al., 2008).

### Statistical Analysis

Significant differences reported in this study were calculated by chi-square test, with the standard significance level setting (^∗∗∗^*P*-value < 0.01, ^∗∗^*P*-value < 0.05).

## Results

### Tracking miRNA Processing Signals by Degradome-Seq Data

In this study, the degradome-seq data of 11 plant species and 4 animal species was retrieved from the public databases (Supplementary Table S1). After pre-treatment (see the Section “Materials and Methods”), the degradome signatures were mapped onto the miRNA precursors obtained from miRBase (release 21) (Kozomara and Griffiths-Jones, 2014), and the perfectly mapped signatures were retained. To search for the prominent degradome signals on the miRNA precursors, an algorithm previously used for cleavage signal identification on the miRNA targets was adopted. Notably, the parameter “signal/noise ratio” (≥5 in default) was introduced into this algorithm to eliminate the interference of RNA random decay (see detail in the Section “Materials and Methods”). Then, we checked whether the extracted degradome signals could be mapped to the 5′ ends or the 3′ ends + 1 nt (i.e., 1-nt downstream of the 3′ ends) of the miRBase-registered mature miRNAs on their precursors. The processing of a mature miRNA was regarded to be supported by degradome-seq data, if a prominent degradome signal could be identified at its 5′ end or 3′ end + 1 nt. Accordingly, the processing of the precursor encoding this mature miRNA was also regarded to be degradome-supported. As a result, the degradome supporting ratios of the miRBase-registered miRNA precursors range from 2.23% (Stu) to 57.56% (Zma) (treating the number of the miRBase-registered miRNA precursors as the denominator) among the 15 species investigated (Table 1). The supporting ratios of eight species, including Zma, Ath, Dme, Bdi, Gma, Sly, Cel, and Osa, are higher than 20%. Four out of the eight species are well-studied model organisms, including two model plants (Ath and Osa) and two model animals (Dme and Cel). Relatively low supporting ratios were observed for human (Hsa, 10.90%) and mouse (Mmu, 7.29%). One of the possible reasons may attribute to the different regulatory mechanisms related to miRNA processing and/or stability between plants and mammals, which results in ineffective detection of processing intermediates by degradome-seq. Additionally, when only treating the number of the precursors with perfectly mapped degradome signatures as the denominator (i.e., removing the precursors without perfectly mapped degradome signature), the supporting ratios were remarkably increased [ranging from 13.16% (Mtr) to 87.69% (Cel)]. And, a total of 13 species achieved the supporting ratios higher than 20%. Moreover, when only treating the number of the precursors with prominent degradome signals as the denominator (i.e., removing the precursors without prominent degradome signal), the supporting ratios were further increased [ranging from 19.61% (Mtr) to 93.44% (Cel)]. A total of 14 species achieved supporting ratios above 25%, and eight species achieved the ratios higher than 50%. In miRBase (release 21), a portion of the precursors were annotated to encode mature miRNAs either on their 5′ (miRNA-5p) or 3′ (miRNA-3p) arms, but not on both arms. Hence, we deduced that some of the prominent degradome signals identified on the precursors might support the processing of the unannotated miRNAs. However, these signals were not included in the above calculation, which could result in the underestimated supporting ratios. Together, the degradome signals show high specificity for detecting miRNA processing intermediates, and the processing of a considerable portion of the miRBase-registered precursors could be tracked by degradome-seq data.

**Table 1:** Specificity of the degradome-seq data for tracking the processing signals of the microRNA precursors registered in miRBase.

Species	A: No. of pre-miRs	HC ratio (%)	B: No. of pre-miRs with perfectly mapped degradome signatures	C: No. of pre-miRs with prominent degradome signals	D: No. of pre-miRs whose processing was supported by the prominent degradome signals	Ratio (D/A%)	Ratio (D/B%)	Ratio (D/C%)
*Arabidopsis thaliana*	325	24	314	312 (306)	172 (151)	52.92	54.78	55.13 (49.35)
*Brachypodium distachyon*	317	NA	193	171 (156)	99 (90)	31.23	51.30	57.89 (57.69)
*Caenorhabditis elegans*	255	44.31	65	61 (59)	57 (55)	22.35	87.69	93.44 (93.22)
*Drosophila melanogaster*	256	58.59	132	115 (94)	97 (77)	37.89	73.48	84.35 (81.91)
*Glycine max*	573	NA	572	369 (339)	174 (151)	30.37	30.42	47.15 (44.54)
*Homo sapiens*	1881	15.74	451	369 (324)	205 (170)	10.90	45.45	55.55 (52.47)
*Medicago truncatula*	672	NA	532	357 (283)	70 (49)	10.42	13.16	19.61 (17.31)
*Mus musculus*	1193	33.95	117	109 (109)	87 (87)	7.29	74.36	79.82 (79.82)
*Oryza sativa*	592	20.44	430	284 (239)	129 (104)	21.79	30.00	45.42 (43.51)
*Physcomitrella patens*	229	42.36	154	103 (66)	42 (32)	18.34	27.27	40.78 (48.48)
*Prunus persica*	180	NA	38	27 (20)	7 (4)	3.89	18.42	25.93 (20.00)
*Solanum lycopersicum*	77	38.96	52	39 (36)	18 (18)	23.38	34.62	46.15 (50.00)
*Solanum tuberosum*	224	3.57	21	20 (20)	5 (5)	2.23	23.81	25.00 (25.00)
*Vitis vinifera*	163	22.09	73	43 (43)	26 (26)	15.95	35.62	60.47 (60.47)
*Zea mays*	172	22.67	160	152 (143)	99 (90)	57.56	61.88	65.13 (62.94)


*The statistical results in parentheses were obtained by adopting the parameter “signal/noise ≥ 50,” and the other results by using the default parameter “signal/noise ≥ 5.” Pre-miR, microRNA precursors registered in miRBase (release 21); HC, high-confidence; NA, not available according to the miRBase annotations.*

As introduced above, to eliminate the random decay noise, the signal/noise ratio was introduced into the algorithm to search for the prominent degradome signals. It is necessary to inspect whether the above result will be greatly influenced by different parameter settings. To address this issue, the signal/noise ratio was increased from “≥5” to “≥50.” Then, the analysis was performed again. The result showed that both the number of the precursors with prominent degradome signals and the number of the precursors whose processing was supported by the prominent degradome signals were slightly decreased in 12 species, leaving the other three species unchanged (Table 1, values in parentheses). Notably, when treating the numbers of the precursors with prominent degradome signals as the denominator, no significant difference was observed between the supporting ratios calculated by adopting the parameter “signal/noise ≥ 5” and “signal/noise ≥ 50” in each species (χ^2^ test, *p* < 0.05). The result indicates that the prominent degradome signals supporting miRNA processing are strong enough to distinguish them from the random degradation background.

**FIGURE 1:**
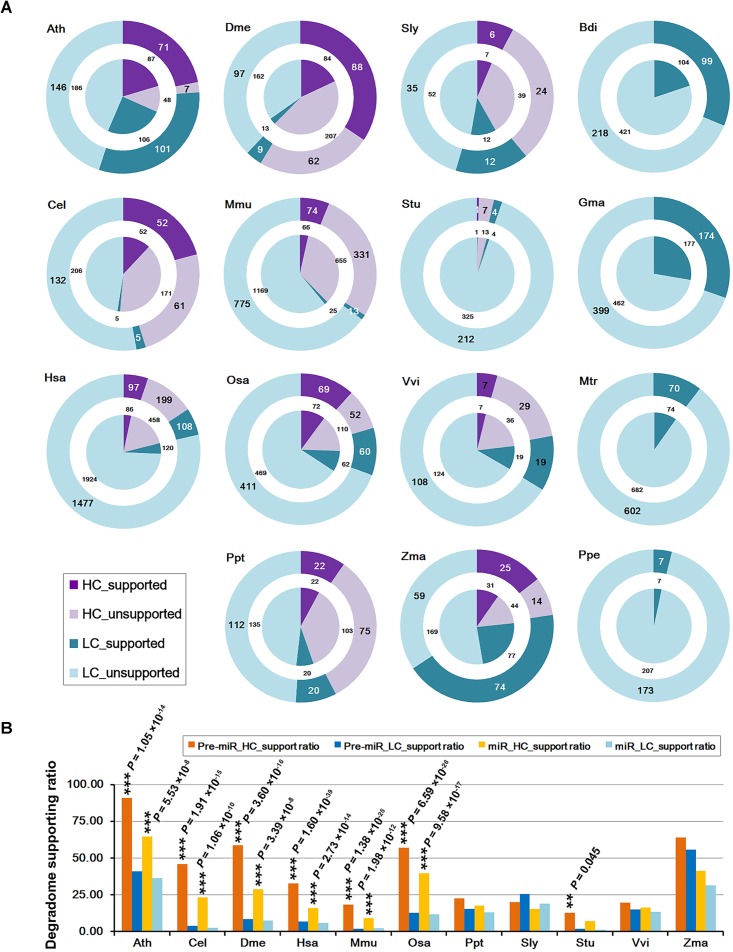
Statistical results of the microRNA (miRNA) genes whose processing was supported by degradome sequencing (degradome-seq) data in 15 species. Generally, the supporting ratios of the high-confidence miRNAs are much higher than those of the low-confidence ones. **(A)** Numbers of the miRNA precursors and the mature miRNAs whose processing was supported by degradome-seq. According to the miRBase annotations (release 21), both the precursors and the miRNAs were classified into “HC” (high-confidence; purple) and “LC” (low-confidence; cyan) categories, respectively. The “supported” and “unsupported” ones were represented by dark and light color separately. The outer rings indicate the precursors while the center pies indicate the mature miRNAs. Four species (Bdi, Gma, Mtr, and Ppe) did not have confidence annotations, and all of the precursors and the mature miRNAs were regarded as “LC” ones here. **(B)** Percentages of the precursors and the mature miRNAs whose processing was supported by degradome-seq. Eleven species with confidence annotations were analyzed. Again, both the precursors and the miRNAs were classified into “HC” (yellow bars, and dark for the precursors and light for the miRNAs) and “LC” (blue bars, and dark for the precursors and light for the miRNAs) ones, respectively. The difference of the degradome supporting ratios between the “HC” and the “LC” categories was examined by χ^2^ test. ^∗∗∗^*P*-value < 0.01, ^∗∗^*P-*value < 0.05. Ath, *Arabidopsis thaliana*; Bdi, *Brachypodium distachyon*; Cel, *Caenorhabditis elegans*; Dme, *Drosophila melanogaster*; Gma, *Glycine max*; Hsa, *Homo sapiens*; Mtr, *Medicago truncatula*; Mmu, *Mus musculus*; Osa, *Oryza sativa*; Ppt, *Physcomitrella patens*; Ppe, *Prunus persica*; Sly, *Solanum lycopersicum*; Stu, *Solanum tuberosum*; Vvi, *Vitis vinifera*; Zma, *Zea mays*.

The “high-confidence” (HC) annotations of both mature miRNAs and the precursors are available in miRBase (release 21) for 11 out of the 15 species investigated in this study (Table 1). The remaining miRNAs precursors without HC annotations were provisionally regarded as the “low-confidence” (LC) ones. Then, the numbers of the miRNAs and the precursors whose processing was supported by degradome-seq were counted for both the HC and the LC categories (Figure 1A). Similar to the above analysis, if there was one prominent degradome signal that could be mapped to either end of the mature miRNA on a precursor, the processing of both the miRNA and its precursor was regarded to be supported by degradome-seq data. Again, the result showed that a considerable portion of both HC and LC categories were supported by degradome-seq data. Deep investigation was performed by calculating the supporting ratios for the 11 species with HC annotations. Notably, in six model species (Ath, Cel, Dme, Hsa, Mmu, and Osa), the supporting ratios of the HC miRNAs were significantly higher than those of the LC ones (χ^2^ test, *p* < 0.01) (Figure 1B). For the four species (Ppt, Sly, Vvi, and Zma) with less-well-studied miRNA populations, no significant difference was observed between the HC miRNAs and the LC ones.

### Distribution of the miRNA Processing Signals

**FIGURE 2:**
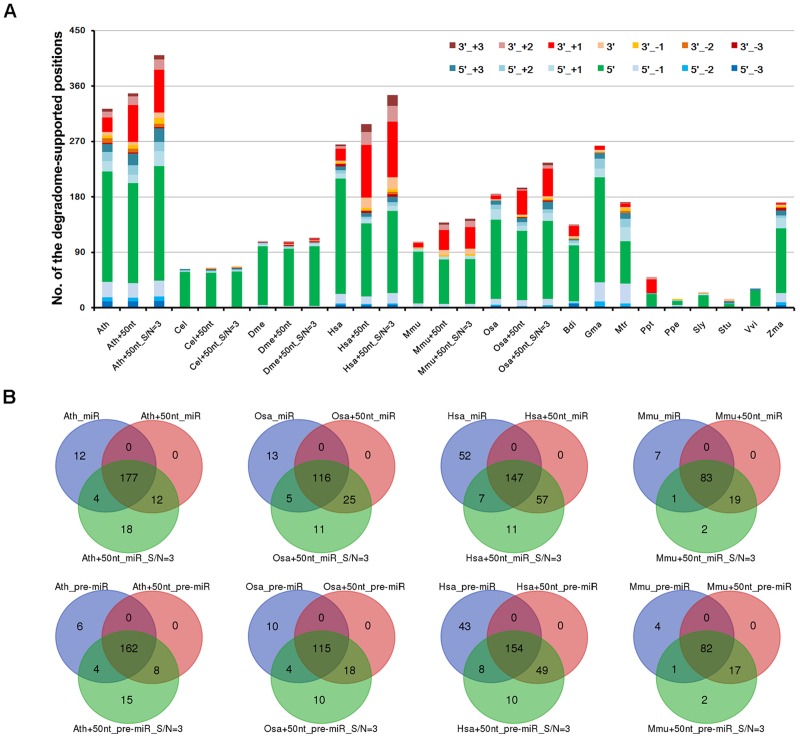
Distribution patterns of the degradome signals on the microRNA (miRNA) precursors. **(A)** A total of 14 positions were examined for each precursors, including 5′ and 3′ ends (“5′” and “3′”) of the mature miRNAs, 1 to 3 nt upstream of the two ends (“5′_+1,” “3′_+1,” “5′_+2,” “3′_+2,” “5′_+3” and “3′_+3”), and 1 to 3 nt downstream of the two ends (“5′_-1,” “3′_-1,” “5′_-2,” “3′_-2,” “5′_-3” and “3′_-3”). Taking *Arabidopsis thaliana* as an example, “Ath” indicates the result of analyzing the miRBase-registered precursors by using the parameter “signal/noise ≥ 5.” “Ath+50nt” indicates the result of analyzing the miRBase-registered precursors with the 50-nt 3′ extensions by using the parameter “signal/noise ≥ 5.” “Ath+50nt_S/N = 3” indicates the result of analyzing the miRBase-registered precursors with the 50-nt 3′ extensions by using the parameter “signal/noise ≥ 3.” The *y*-axis measures the numbers of the positions supported by degradome signals. **(B)** Venn diagrams showing the intersections of the degradome-supported mature miRNAs and precursors identified from three independent analyses in four species. Taking *Arabidopsis thaliana* as an example, “Ath_miR” and “Ath_pre-miR” indicate the degradome-supported miRNAs and precursors identified by analyzing the miRBase-registered precursors using “signal/noise ratio ≥ 5.” “Ath+50nt_miR” and “Ath+50nt_pre-miR” indicate the degradome-supported miRNAs and precursors identified by analyzing the precursors with the 50-nt 3′ extensions using “signal/noise ratio ≥ 5.” “Ath+50nt_miR_S/N = 3” and “Ath+50nt_pre-miR_S/N = 3” indicate the degradome-supported miRNAs and precursors identified by analyzing the precursors with the 50-nt 3′ extensions using “signal/noise ratio ≥ 3.” Ath, *Arabidopsis thaliana*; Bdi, *Brachypodium distachyon*; Cel, *Caenorhabditis elegans*; Dme, *Drosophila melanogaster*; Gma, *Glycine max*; Hsa, *Homo sapiens*; Mtr, *Medicago truncatula*; Mmu, *Mus musculus*; Osa, *Oryza sativa*; Ppt, *Physcomitrella patens*; Ppe, *Prunus persica*; Sly, *Solanum lycopersicum*; Stu, *Solanum tuberosum*; Vvi, *Vitis vinifera*; Zma, *Zea mays*.

To view the distribution pattern of the degradome signals supporting miRNA processing, the processing sites on the precursors were classified into two categories, i.e., the 5′ ends and the 3′ ends + 1 nt of the mature miRNAs. For clarity, the two positions were named as 5′ and 3′ processing sites, respectively. The numbers of the two sites supported by the prominent degradome signals were counted for each species. At first glance, the processing signals detected by degradome-seq are highly enriched at the 5′ processing sites, which is highly consistent among the 15 species (Figure 2A). Since the degradome signatures of 20 nt or longer might be failed in mapping to the short downstream regions of the 3′-armed miRNAs, the 50-nt 3′ extensions of the miRNA precursors were collected from the genomes of the six model species (Ath, Cel, Dme, Hsa, Mmu, and Osa). Then, the extended precursors were subject to degradome mapping and prominent signal search. As a result, the numbers of the 3′ processing sites supported by degradome-seq data were greatly increased in the four species (from 24 to 60 in Ath, from 19 to 85 in Hsa, from 7 to 32 in Mmu, and from 6 to 39 in Osa) (Figure 2A). Strikingly, none was detected in Cel, and only two 3′ processing sites were supported by the degradome-seq data in Dme. For the four model species including Ath, Osa, Hsa, and Mmu, three independent analyses were performed: (1) Mapping degradome-seq data onto the miRNA precursors, and using the parameter “signal/noise ≥ 5.” (2) Mapping degradome-seq data onto the precursors with 50-nt 3′ extensions, and using “signal/noise ≥ 5.” (3) Mapping degradome-seq data onto the precursors with 50-nt 3′ extensions, and using “signal/noise ≥ 3.” As a result, great intersections were observed among the three independent analyses for all of the species analyzed (Figure 2B), which pointed to the high sensitivity and specificity of degradome-seq data in tracking miRNA processing signals. Another notable observation was that a portion of the prominent degradome signals were mapped to the neighboring positions of the 5′ or the 3′ processing sites (Figure 2A), which reminiscently indicated isomiR production reported in both plants and animals (Cloonan et al., 2011; Neilsen et al., 2012; Budak et al., 2015).

To gain a deeper view, the processing sites on a precursor were further assigned to four positions, including the 5′ end of the 5′-armed miRNA (called position “1” hereafter), the 3′ end + 1 nt of the 5′-armed miRNA (position “2”), the 5′ end of the 3′-armed miRNA (position “3”), and the 3′ end + 1 nt of the 3′-armed miRNA (position “4”). Then, the percentage of each position detected with degradome signals was calculated. As a result, enrichment of the degradome-supported processing signals was observed at the 5′ ends of the mature miRNAs, which was conserved between plants and animals. In other words, the percentages of the positions “1” and “3” were much higher than those of the positions “2” and “4.” Then, the percentages were calculated again after recruitment of the 50-nt 3′ extensions of the miRNA precursors. Compared to the first result, the percentages of the position “4” were greatly elevated in Ath, Hsa, Mmu and Osa, except for Cel and Dme (Supplementary Figure S1). However, in both cases, the percentages of the position “2” are very low in all species analyzed. According to the miRBase annotations of the 15 species analyzed, the number of the 5′-armed miRNAs is nearly equal to that of the 3′-armed ones. Thus, the low percentages of the position “2” detected with prominent degradome signals were not likely to be caused by the distribution bias of the mature miRNAs on the two arms of the precursors.

### Degradome Supporting Ratios Increased With Sampling Diversity

**FIGURE 3:**
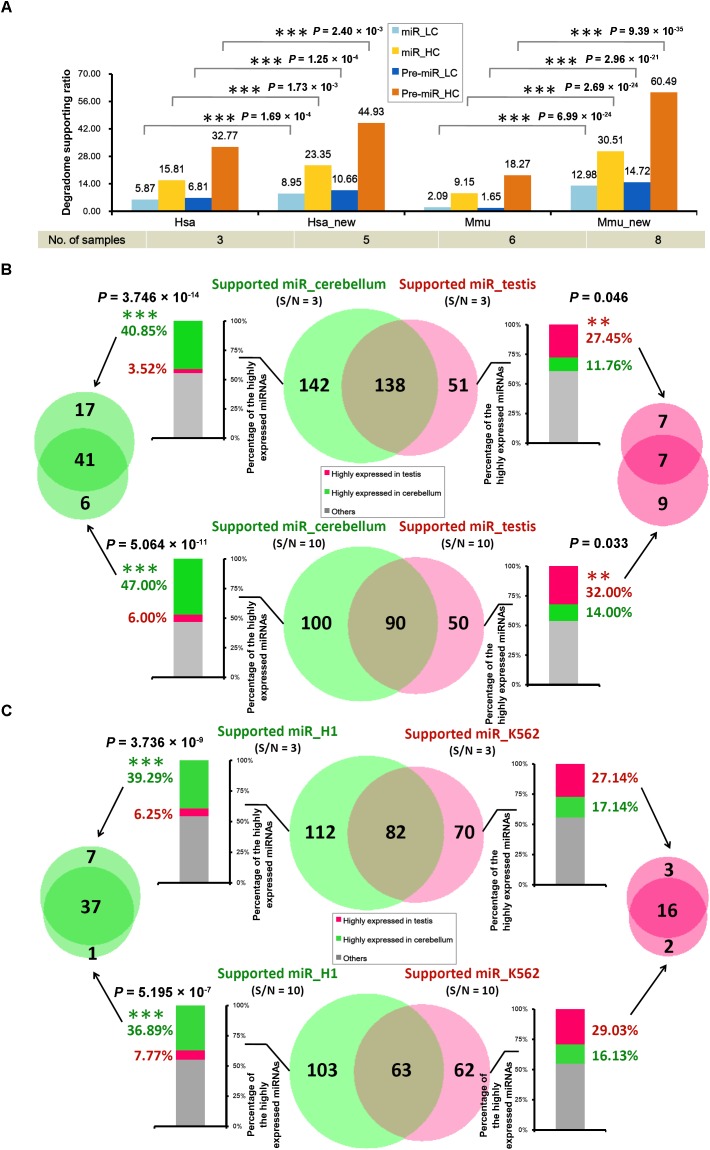
The tissue- or cell line-specific processing patterns of the microRNA (miRNA) precursors partially contributed to the accumulation patterns of the mature miRNAs. **(A)** Supporting ratios of miRNA processing (measured by the *y*-axis of the histogram) were increased with degradome sampling diversity (listed in the table below the histogram). miR, mature miRNAs; Pre-miR, miRNA precursors; HC, high-confidence registries in miRBase (release 21); LC, low-confidence registries; Hsa, the first round of analysis in *Homo sapiens*; Hsa_new, the second round of analysis in *Homo sapiens* by adding new datasets of degradome-seq; Mmu, the first round of analysis in *Mus musculus*; Mmu_new, the second round of analysis in *Mus musculus* by adding new datasets of degradome-seq. See detail of these datasets in Supplementary Table S1. **(B)** In mouse, degradome-seq datasets from cerebellum and testis were separately used to identify the degradome-supported mature miRNAs. The parameter “signal/noise ratio” (S/N) was adjusted to be ≥3 and ≥10, respectively, for the analysis. The center Venn diagrams indicate that 142 and 100 miRNAs were found to be specifically supported by the cerebellum-derived degradome-seq data under S/N ≥ 3 and 10, respectively. And, 51 and 50 miRNAs were specifically supported by the testis-derived degradome data under S/N ≥ 3 and 10, respectively. The stacked histograms on the left show the expression patterns of the miRNAs specifically supported by the cerebellum-derived degradome-seq data. And, the histograms on the right show the expression patterns of the miRNAs specifically supported by the testis-derived degradome data. The *y*-axes measure the percentages of the highly expressed miRNAs (green: highly expressed in cerebellum; red: highly expressed in testis). The Venn diagram on the left indicates that 41 miRNAs highly expressed in the cerebellum could be detected by adopting both S/N ≥ 3 and ≥10. The Venn diagram on the right indicates that seven miRNAs highly expressed in the testis could be detected by adopting either of the two parameters. **(C)** Degradome-seq datasets from human cell lines H1 and K562 were separately used to identify the degradome-supported miRNAs. The analysis was similar to that applied to mouse. χ^2^ test significance levels: ^∗∗∗^*P*-value < 0.01, ^∗∗^*P*-value < 0.05.

It was observed that only 205 out of the 1,881 miRNA precursors (10.90%) in human, and 87 out of the 1,193 precursors (7.29%) in mouse were detected with degradome-supported processing signals (Table 1). This result was calculated based on the degradome-seq data prepared from brain, HeLa cell line and H1 cell line of human, and the data prepared from brain, lung, liver, kidney, ovary, and spleen of mouse. Fortunately, additional degradome-seq data of different sample resources (including HEK293 and K562 cell lines of human, and cerebellum and testis of mouse) was obtained for the two species (Supplementary Table S1). We wondered whether the ratios of the degradome-supported miRNA precursors could be affected by sampling diversity. To this end, the parameter (signal/noise ≥ 5) identical to the first round of analysis was used to search for the prominent signals by using all of the available degradome-seq data. Then, the degradome signals were mapped to the ends of the mature miRNA-coding regions, in order to calculate the degradome supporting ratios. Notably, significant increments of the supporting ratios were observed for both the HC and the LC categories in the two species (χ^2^ test, *p* < 0.01) (Figure 3A and Supplementary Figure S1). In human, the ratios of the HC miRNAs and the HC precursors with degradome-supported processing signals were increased from 15.81% to 23.35% and from 32.77% to 44.93%, respectively. In mouse, the degradome supporting ratios were increased from 9.15% to 30.51% and from 18.27% to 60.49%, respectively. On the other hand, the supporting ratios of the LC miRNAs and precursors were increased from 5.87% to 8.95% and from 6.81% to 10.66% in human, respectively, and from 2.09% to 12.98% and from 1.65% to 14.72% in mouse, respectively. Together, it demonstrated that the increased sampling diversity of the degradome-seq data (from three to five samples in human, and from six to eight in mouse) could significantly elevate the ratios of the miRNAs with degradome-supported processing signals. Thus, the relatively low supporting ratios observed during the first round of analysis were likely to be caused by unsaturated sampling. From another point of view, huge degradome-seq datasets with diversified biological origins were desired for tracking the miRNA processing signals.

### Partial Relationship Between miRNA Processing and Accumulation

According to the current model of miRNA biogenesis and action, the accumulation levels of the mature miRNAs are affected by many other factors (Meng et al., 2011), including the processing procedure. The above result indicates that a portion of the miRNAs are processed with tissue- or cell line-specific patterns in both human and mouse. Thus, we wondered whether the processing patterns of the miRNAs could contribute to, at least partially, their accumulation patterns.

In mouse, the degradome signatures from cerebellum and testis were mapped onto the miRNA precursors with 50-nt 3′ extensions. Two different parameter settings, i.e., signal/noise “≥3” and “≥10,” were used to search for the prominent degradome signals, respectively. Then, the degradome signals were mapped to the 5′ and the 3′ ends of the mature miRNAs, in order to identify the miRNAs with degradome-supported processing signals. As a result, 280 and 189 miRNAs were extracted from the analysis of cerebellum- and testis-originated degradome data, respectively, by adopting “signal/noise ≥ 3” (Figure 3B). After removing the intersection (138), 142 miRNAs were specifically supported by the cerebellum degradome, and 51 miRNAs were specifically supported by the testis degradome. The more stringent parameter “signal/noise ≥ 10” resulted in the smaller numbers of the identified miRNAs, including 100 and 50 miRNAs specifically supported by the cerebellum- and the testis-originated degradome data, respectively. Then, sRNA-seq data from the two tissues was used to inspect the accumulation levels of the miRNAs identified above. If the level of a miRNA was higher than 1 RPM in tissue A, and was two times or higher than that in tissue B, then the miRNA was considered to be highly accumulated in tissue A. For “signal/noise ≥ 3,” 58 out of the 142 miRNAs (40.85%) whose processing was specifically supported by the cerebellum degradome were highly accumulated in cerebellum, while only five miRNAs (3.52%) were highly accumulated in testis. And, 14 out of the 51 miRNAs (27.45%) whose processing was specifically supported by the testis degradome were highly accumulated in the testis, while only six miRNAs (11.76%) were highly accumulated in cerebellum (Figure 3B and Supplementary Table S2). A similar scene was observed for “signal/noise ≥ 10.” Specifically, 47 out of the 100 miRNAs (47.00%) whose processing was specifically supported by the cerebellum degradome were highly accumulated in cerebellum, while only six miRNAs (6.00%) were highly accumulated in testis. And, 16 out of the 50 miRNAs (32.00%) whose processing was specifically supported by the testis degradome were highly accumulated in testis, while only seven miRNAs (14.00%) were highly accumulated in cerebellum (Figure 3B and Supplementary Table S2).

In human, similar analysis was performed by using degradome-seq and sRNA-seq data prepared from H1 and K562 cell lines. For “signal/noise ≥ 3,” 44 out of the 112 miRNAs (39.29%) whose processing was specifically supported by the H1 degradome were highly accumulated in H1, while only seven miRNAs (6.25%) were highly accumulated in K562. And, 19 out of the 70 miRNAs (27.14%) whose processing was specifically supported by the K562 degradome were highly accumulated in K562, while only 12 miRNAs (17.14%) were highly accumulated in H1 (Figure 3C and Supplementary Table S3). For “signal/noise ≥ 10,” 38 out of the 103 miRNAs (36.89%) whose processing was specifically supported by the H1 degradome were highly accumulated in H1, while only eight miRNAs (7.77%) were highly accumulated in K562. And, 18 out of the 62 miRNAs (29.03%) whose processing was specifically supported by the K562 degradome were highly accumulated in K562, while only ten miRNAs (16.13%) were highly accumulated in H1 (Figure 3C and Supplementary Table S3).

Taken together, the above results reach the conclusion that the tissue- or cell line-specific processing patterns of the miRNAs partially contribute to their accumulation patterns.

### Annotating miRNAs Based on Degradome-Seq Data

Given the high specificity and sensitivity of degradome-seq data in tracking miRNA processing signals, it was interesting to see the ability of degradome signatures in re-examination of the miRBase registries, or in the discovery of novel miRNAs. Fortunately, several exquisite examples showed the capacity of degradome-seq data in miRNA annotation.

**FIGURE 4:**
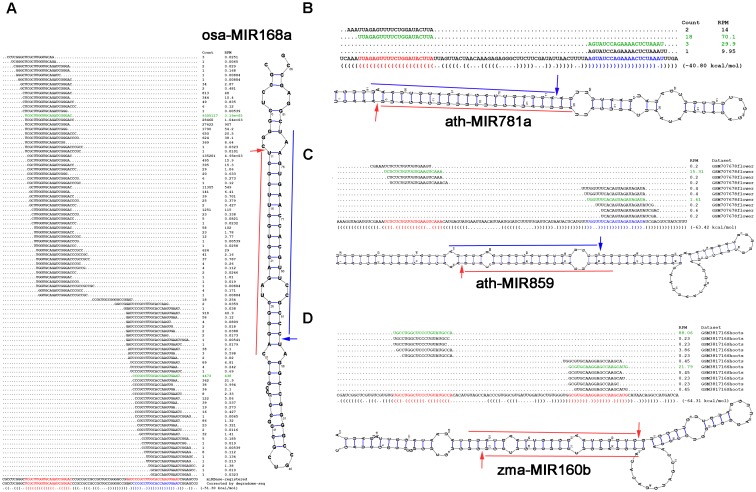
Examples showing the value of degradome sequencing (degradome-seq) data for microRNA (miRNA) re-annotation. Based on degradome-seq and small RNA sequencing (sRNA-seq) data, and secondary structures, **(A)** the sequence of osa-miR168a-3p (GAUCCCGCCUUGCACCAAGUGAAU; red color) registered in miRBase (release 21) was recommended to be corrected as “CCCGCCUUGCACCAAGUGAAU” (blue color), novel miRNAs (blue color) located on the 3′ arms of **(B)** ath-MIR781a and **(C)** ath-MIR859 were identified, and **(D)** both of the miRBase-registered miRNAs on the precursor zma-MIR160b were suggested to be the high-confidence candidates. For each panel, the sRNA mapping result (including expression levels in RPM, reads per million), either retrieved from miRBase or based on the analysis of the public dataset, is shown above the sequence of the precursor. The dot-bracket notation below the precursor correlates with the secondary structure predicted by RNAshapes. The expression evidence of the mature miRNAs are highlighted in green color, and the processing sites supported by the degradome signals are denoted by arrows on the predicted secondary structure of the miRNA precursor.

In rice (Osa), the precursor osa-MIR168a forms a short stem-loop structure. According to the miRBase annotation (release 21), osa-miR168a-5p (UCGCUUGGUGCAGAUCGGGAC) and osa-miR168a-3p (GAUCCCGCCUUGCACCAAGUGAAU) are encoded on the 5′ and 3′ arms of the precursor, respectively (Figure 4A). One of the degradome signals discovered on this precursor could be mapped to the 5′ processing site of osa-miR168a-5p, indicating its high confidence. However, no signal supports the processing of osa-miR168a-3p. Instead, another prominent degradome signal was mapped to the position between the third and the fourth nucleotides of osa-miR168a-3p, indicating that the sequence “CCCGCCUUGCACCAAGUGAAU,” 3-nt shorter from the 5′ end of osa-miR168a-3p, might be a stronger candidate. In plants, DCL1-mediated processing will result in 2-nt 3′ overhangs of the miRNA-5p:miRNA-3p duplexes (Voinnet, 2009). Based on the secondary structure of osa-MIR168a, the sequence “CCCGCCUUGCACCAAGUGAAU,” but not osa-miR168a-3p, could form a short duplex with osa-miR168a-5p, fulfilling the “2-nt 3′ overhang” criterion. Further evidence was achieved from the accumulation levels of the sRNAs mapped onto the precursor. Two sRNA clusters were discovered on the 5′ and the 3′ arms of the precursor, respectively. Within the 5′-armed cluster, osa-miR168a-5p was expressed at the highest level. However, within the 3′ cluster, the sequence “CCCGCCUUGCACCAAGUGAAU,” but not osa-miR168a-3p, was most abundantly accumulated. The above three pieces of evidence strongly recommended that the sequence of osa-miR168a-3p might be re-annotated as “CCCGCCUUGCACCAAGUGAAU.”

In Arabidopsis (Ath), only 5′-armed miRNAs were annotated on the two precursors ath-MIR781a and ath-MIR859. The processing of both 5′-armed miRNAs was supported by the degradome signals (Figures 4B,C). Based on secondary structure prediction and degradome signal mapping, the 3′-armed miRNA candidates were identified to form 2-nt 3′ overhanged duplexes with the 5′-armed miRNAs. Further evidence was obtained from sRNA expression data that both of the 3′-armed candidates were accumulated at high levels within the 3′ sRNA clusters on their precursors.

In maize (Zma), both zma-miR160b-5p and zma-miR160b-3p were annotated on the precursor zma-MIR160b, but were not annotated as the HC ones (miRBase, release 21). However, degradome signals could be mapped to the 5′ processing sites of both miRNAs. The two miRNAs could form a short duplex with 2-nt 3′ overhangs (Figure 4D). Besides, within the 5′- and the 3′ sRNA clusters, both of the miRNAs were accumulated at the highest levels, respectively. In this regard, these two miRNAs might be annotated as the HC ones.

Taken together, the above examples indicate that by combining with secondary structure prediction and sRNA-seq data analysis, degradome-seq data is especially valuable for re-examination of the miRNA registries, and identification of novel miRNA candidates.

### Sequence Motifs Surrounding the Processing Sites

Given the fact that analogous hairpin structures are widespread in a cell, it is amazing that the stem regions of the miRNA precursors can be specifically recognized by animal Drosha/Dicer or plant DCL1 for miRNA maturation. Although a few research efforts have been taken to decipher the linear or secondary codes for miRNA processing (Werner et al., 2010; Roden et al., 2017), it is still far from thorough decoding. Here, we searched for the sequence motifs surrounding the processing sites, which might contribute to miRNA processing. To this end, four species (Ath, Osa, Hsa, and Mmu) were analyzed by using all of the available degradome-seq data. The degradome signatures were mapped onto the miRNA precursors with 50-nt 3′ extensions. The parameter “signal/noise ≥ 3” was adopted for degradome signal search. As mentioned above, the processing sites on a precursor were classified into four positions, i.e., “1,” “2,” “3,” and “4.” Consistent with the above results, the degradome signals were highly enriched at the positions “1,” “3,” and “4” of the four species. For all of the miRNA precursors analyzed in Arabidopsis (Ath), a total of 85, 101, and 67 degradome-supported processing sites were assigned to the positions “1,” “3,” and “4,” respectively. In rice (Osa), 72, 55, and 43 degradome-supported processing sites were assigned to “1,” “3,” and “4,” respectively. In human (Hsa), 133, 86, and 135 degradome-supported processing sites were assigned to “1,” “3,” and “4,” respectively. In mouse (Mmu), 184, 130, and 98 degradome-supported processing sites were assigned to “1,” “3,” and “4,” respectively. Then, the 20-nt sequences surrounding (10-nt upstream and 10-nt downstream) the degradome-supported processing sites were collected, and subject to sequence motif prediction. The 20-nt sequences surrounding the processing sites “1,” “3,” and “4” that were not supported by degradome signals served as the control sets.

**FIGURE 5:**
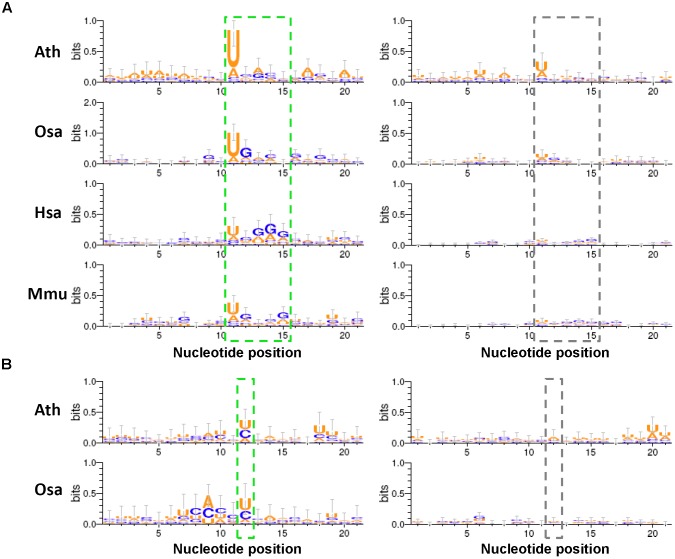
Sequence motifs predicted within the regions surrounding the degradome-supported processing sites of the microRNA (miRNA) precursors. **(A)** The short regions (marked by a green dashed box) near the degradome-supported 5′ ends of the 5′-armed microRNAs (miRNAs) show relatively high sequence conservation, compared to the control sets (marked by a gray dashed box). **(B)** In the two model plants, the first nucleotides (marked by a green dashed box) downstream of the 3′ ends of the 3′-armed miRNAs show a strong “U/C” bias, compared to the control sets (marked by a gray dashed box). The sequence conservation diagrams were drawn by WebLogo 3. The *x*-axes indicate the nucleotide positions, and the 5′ ends of the 5′-armed miRNAs as mentioned in **(A)** and the first nucleotides downstream of the 3′ ends of the 3′-armed miRNAs as mentioned in **(B)** were both assigned to the 11th nucleotides on the *x*-axes. The *y*-axes measure the degree of sequence conservation. Ath, *Arabidopsis thaliana*; Osa, *Oryza sativa*; Hsa, *Homo sapiens*; Mmu, *Mus musculus*.

As a result, some interesting motifs were detected within the 20-nt regions surrounding the processing sites “1” and “4.” Compared to the control sets, the degradome-supported processing sites “1,” i.e., the 5′ ends of the 5′-armed miRNAs, showed a strong preference for “U” in all of the four species analyzed (Figure 5A). Notably, 5′ U was reported to be preferred by the canonical miRNAs incorporated into Argonaute 1 in plants (Mi et al., 2008). Additionally, a weak enrichment of “G” was observed within the 4-nt downstream regions of the degradome-supported processing sites “1” in rice, human and mouse. Another interesting motif “U/C” was observed for the first nucleotide downstream of the degradome-supported processing sites “4” in Arabidopsis and rice (Figure 5B). Together, whether these specious motifs are related to the miRNA processing codes remains to be further investigated.

## Discussion

### Degradome-Seq Data: A Valuable Resource for miRNA Annotations

In this study, the algorithm previously used to cleavage site search on the miRNA targets (German et al., 2008; Shao et al., 2013) was adopted to identify the processing signals on the miRNA precursors. By introducing the parameter “signal/noise” ratio, the algorithm showed its high sensitivity and specificity for tracking the miRNA processing intermediates based on the degradome-seq data (Table 1, Figure 2, and Supplementary Figure S1). Integrative analysis of secondary structures, degradome-seq data and sRNA-seq data results in several exquisite examples (Figure 4), indicating the value of degradome-seq data in miRNA annotation. Furthermore, the ratio of the miRNAs with degradome-supporting processing signals was observed to be much higher for the HC miRNAs annotated in miRBase (release 21), compared to the LC ones (Figure 1B). Thus, the degradome-based analysis might facilitate the “confidence” annotation of the current miRNA registries. Additionally, our results indicated that the degradome-supported, spatio-temporally specific processing patterns of the miRNA precursors could partially reflect the accumulation patterns of the mature miRNAs.

### A Renewed Model of miRNA Processing

**FIGURE 6:**
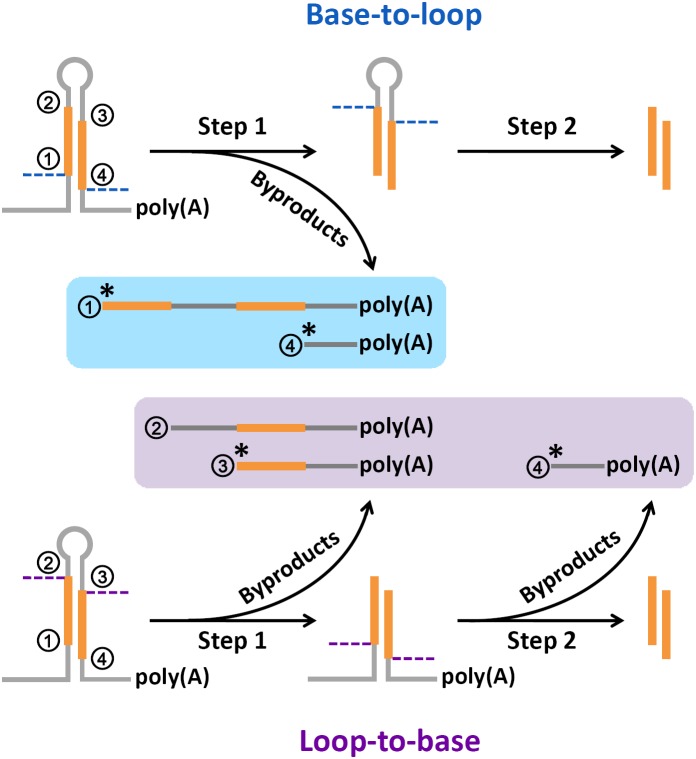
A renewed model proposed for microRNA (miRNA) processing. Based on the abundant degradome signals at position “1,” the single-stranded cropping mode (i.e., the double-stranded stems were cropped through one strand at a time not two strands at a time) was preferred during “base-to-loop” processing. And, based on the abundant degradome signals at position “3,” “loop-to-base” or bidirectional processing was suggested to be occurred more frequently than previously thought. “^∗^” indicates a high degradome supporting ratio at the marked processing sites.

In this study, high degradome supporting ratios were observed at the processing sites “1,” “3,” and “4” on the miRNA precursors of Arabidopsis, rice, human, and mouse (Supplementary Figure S1). To date, three major modes have been proposed for miRNA processing, including “base-to-loop,” “loop-to-base” and bidirectional processing (Bologna et al., 2009; Schwab and Voinnet, 2009; Zhu et al., 2013; Zhang et al., 2015). However, in both plants and animals, it is still unclear whether the Drosha/Dicer- or DCL1-mediated processing on the stem regions was performed through the single- or double-stranded cropping mode. Our study showed that the polyadenylated processing intermediates containing the processing sites “1” were highly abundant, which was reflected by the degradome signals. Notably, these abundant intermediates could only be produced through the single-stranded cropping mode (i.e., cropped through one strand at a time) during “base-to-loop” processing (Figure 6). A method called SPARE, derived from degradome-seq, was developed for specific detection of miRNA processing intermediates (Bologna et al., 2013; Schapire et al., 2013). In this study, the publicly available SPARE data of Arabidopsis was analyzed to check the enrichment of degradome signals at the processing sites “1.” Expectedly, the high SPARE supporting ratio was observed at the processing sites “1” in Arabidopsis (Supplementary Figure S2).

Additionally, the polyadenylated processing intermediates containing the processing sites “3” were also abundant based on our results of degradome-seq data analysis. This type of remnants could only be generated during “loop-to-base” processing (Figure 6). Although “base-to-loop” processing was previously considered to be the dominant mode for miRNA maturation (Schwab and Voinnet, 2009; Zhang et al., 2015), the degradome signals indicated that “loop-to-base” or bidirectional processing might occur more frequently than previously thought. Besides, the analysis of SPARE data also showed a high SPARE supporting ratio at the processing sites “3” in Arabidopsis (Supplementary Figure S2).

Summarily, a renewed model of miRNA processing is proposed that in many cases, the stem regions of the precursors are diced through a “single-stranded cropping” mode, and “loop-to-base” processing may occur more frequently than previously thought. However, this model needs further experimental validation.

## Conclusion

In this study, by introducing an algorithm with the adjustable parameter “signal/noise” ratio, the prominent degradome signals were identified on the miRNA precursors of 15 different species. A large portion of these signals could be mapped to the processing sites at the ends of the miRNA-coding regions, unraveling the value of degradome-seq data in tracking miRNA processing intermediates in both plants and animals. Besides, we demonstrated that the tissue- or cell line-specific processing patterns of the miRNA precursors partially contributed to the accumulation patterns of the mature miRNAs. Based on the distribution patterns of the degradome signals on the precursors, a renewed model was proposed for miRNA processing. Taken together, we hope that our study could advance the current knowledge on the application value of degradome-seq data and the miRNA processing mechanism.

## Author Contributions

YM and HW conceived and designed the experiments. DY, MX, WS, and XM analyzed the data. YM and DY collected sequencing data. YM and HI wrote the paper. All authors read and approved the final manuscript.

## Conflict of Interest Statement

The authors declare that the research was conducted in the absence of any commercial or financial relationships that could be construed as a potential conflict of interest.

## Footnotes

1 http://www.mirbase.org/

2 http://www.arabidopsis.org/

3 ftp://ftp.ensembl.org/pub/release-88/fasta/caenorhabditis_elegans/dna/

4 http://www.fruitfly.org/sequence/release5genomic.shtml

5 https://www.ncbi.nlm.nih.gov/projects/genome/guide/human/#download

6 https://www.ncbi.nlm.nih.gov/genome?term=mus%20musculus

7 http://rice.plantbiology.msu.edu/

8 https://www.ncbi.nlm.nih.gov/geo/

9 https://www.ncbi.nlm.nih.gov/sra/

10 http://mpss.danforthcenter.org/

11 http://bioinformatics.psb.ugent.be/webtools/Venn/

## Abbreviations

Ath: *Arabidopsis thaliana*
Bdi: *Brachypodium distachyon*
Cel: *Caenorhabditis elegans*
DCL1: Dicer-like 1
Degradome-seq: degradome sequencing
Dme: *Drosophila melanogaster*
GEO: Gene Expression Omnibus
Gma: *Glycine max*
GMUCT: genome-wide mapping of uncapped and cleaved transcripts
HC: high-confidence
Hsa: *Homo sapiens*
LC: low-confidence
miRNA: microRNA
Mmu: *Mus musculus*
Mtr: *Medicago truncatula*
Osa: *Oryza sativa*
PARE: parallel analysis of RNA ends
Ppe: *Prunus persica*
Ppt: *Physcomitrella patens*
RPM: reads per million
siRNA: small interfering RNA
Sly: *Solanum lycopersicum*
SPARE: specific parallel amplification of RNA ends
SRA: Sequence Read Archive
sRNA-seq: small RNA sequencing
Stu: *Solanum tuberosum*
Vvi: *Vitis vinifera*
Zma: *Zea mays*
