# Supplementary material for: Tracking microRNA Processing Signals by Degradome Sequencing Data Analysis
Source: Front Genet. 2018 Nov 14;9:546. doi: 10.3389/fgene.2018.00546 (PMC6246748; doi:10.3389/fgene.2018.00546)
Supplement: Supplementary file 1 [file Data_Sheet_1.pdf]

Figure S1 Statistical results showing the degradome supporting ratios at four processing sites on the microRNA (miRNA) precursors of 15 species. The mature miRNAs are represented by yellow lines on the stem-loop precursors. There are four categories of the statistical results. Category I: mapping degradome signatures onto the miRBase-registered precursors, and searching for degradome signals by using the parameter "signal/noise >= 5". Category II: mapping degradome signatures onto the precursors with the 50-nt 3' extensions, and searching for degradome signals by "signal/noise >= 5". Category III: mapping degradome signatures onto the precursors with the 50-nt 3' extensions, and searching for degradome signals by "signal/noise >= 3". Category IV: mapping degradome signatures, including newly added datasets, onto the precursors, and searching for degradome signals by "signal/noise >= 5". T: statistical results for all of the processing sites according to the miRBase annotations (release 21). H: statistical results for the processing sites belonging to the high-confidence mature miRNAs registered in miRBase (release 21). L: statistical results for the processing sites belonging to the non-high-confidence mature miRNAs registered in miRBase (release 21). For some species without "confidence" annotations, only "T" results are shown. Ath: *Arabidopsis thaliana*; Bdi: *Brachypodium distachyon*; Cel: *Caenorhabditis elegans*; Dme: *Drosophila melanogaster*; Gma: *Glycine max*; Hsa: *Homo sapiens*; Mmu: *Mus musculus*; Mtr: *Medicago truncatula*; Osa: *Oryza sativa*; Ppt: *Physcomitrella patens*; Ppe: *Prunus persica*; Sly: *Solanum lycopersicum*; Stu: *Solanum tuberosum*; Vvi: *Vitis vinifera*; Zma: *Zea mays*.

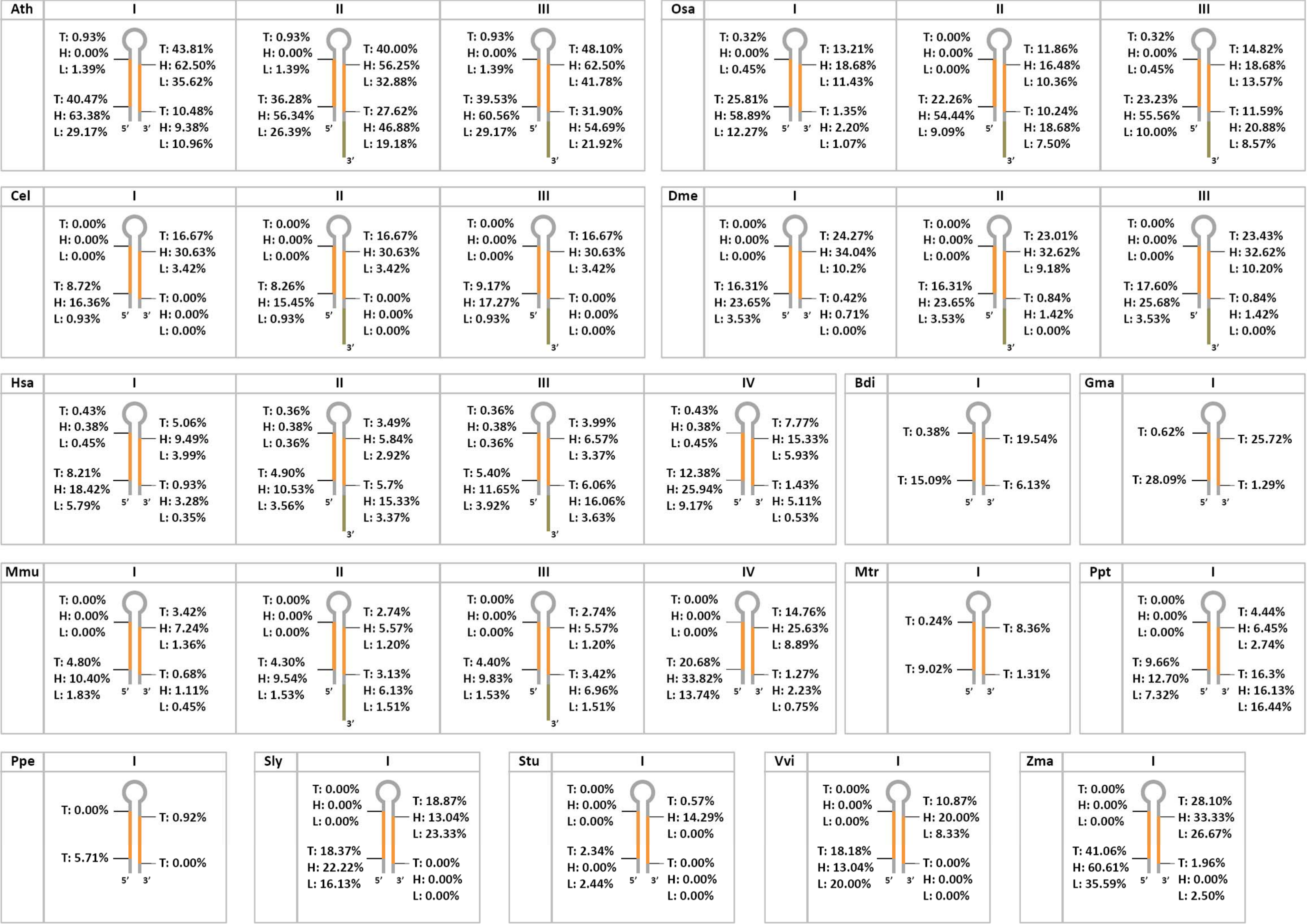

Ath
I
T: 0.93%
H: 0.00%
L: 1.39%
T: 43.81%
H: 62.50%
L: 35.62%
T: 40.47%
H: 63.38%
L: 29.17%
T: 10.48%
H: 9.38%
L: 10.96%
5′ 3′
II
T: 0.93%
H: 0.00%
L: 1.39%
T: 40.00%
H: 56.25%
L: 32.88%
T: 36.28%
H: 56.34%
L: 26.39%
T: 27.62%
H: 46.88%
L: 19.18%
5′ 3′
III
T: 0.93%
H: 0.00%
L: 1.39%
T: 48.10%
H: 62.50%
L: 41.78%
T: 39.53%
H: 60.56%
L: 29.17%
T: 31.90%
H: 54.69%
L: 21.92%
5′ 3′
Osa
I
T: 0.32%
H: 0.00%
L: 0.45%
T: 13.21%
H: 18.68%
L: 11.43%
T: 25.81%
H: 58.89%
L: 12.27%
T: 1.35%
H: 2.20%
L: 1.07%
5′ 3′
II
T: 0.00%
H: 0.00%
L: 0.00%
T: 11.86%
H: 16.48%
L: 10.36%
T: 22.26%
H: 54.44%
L: 9.09%
T: 10.24%
H: 18.68%
L: 7.50%
5′ 3′
III
T: 0.32%
H: 0.00%
L: 0.45%
T: 14.82%
H: 18.68%
L: 13.57%
T: 23.23%
H: 55.56%
L: 10.00%
T: 11.59%
H: 20.88%
L: 8.57%
5′ 3′
Cel
I
T: 0.00%
H: 0.00%
L: 0.00%
T: 16.67%
H: 30.63%
L: 3.42%
T: 8.72%
H: 16.36%
L: 0.93%
T: 0.00%
H: 0.00%
L: 0.00%
5′ 3′
II
T: 0.00%
H: 0.00%
L: 0.00%
T: 16.67%
H: 30.63%
L: 3.42%
T: 8.26%
H: 15.45%
L: 0.93%
T: 0.00%
H: 0.00%
L: 0.00%
5′ 3′
III
T: 0.00%
H: 0.00%
L: 0.00%
T: 16.67%
H: 30.63%
L: 3.42%
T: 9.17%
H: 17.27%
L: 0.93%
T: 0.00%
H: 0.00%
L: 0.00%
5′ 3′
Dme
I
T: 0.00%
H: 0.00%
L: 0.00%
T: 24.27%
H: 34.04%
L: 10.2%
T: 16.31%
H: 23.65%
L: 3.53%
T: 0.42%
H: 0.71%
L: 0.00%
5′ 3′
II
T: 0.00%
H: 0.00%
L: 0.00%
T: 23.01%
H: 32.62%
L: 9.18%
T: 16.31%
H: 23.65%
L: 3.53%
T: 0.84%
H: 1.42%
L: 0.00%
5′ 3′
III
T: 0.00%
H: 0.00%
L: 0.00%
T: 23.43%
H: 32.62%
L: 10.20%
T: 17.60%
H: 25.68%
L: 3.53%
T: 0.84%
H: 1.42%
L: 0.00%
5′ 3′
Hsa
I
T: 0.43%
H: 0.38%
L: 0.45%
T: 5.06%
H: 9.49%
L: 3.99%
T: 8.21%
H: 18.42%
L: 5.79%
T: 0.93%
H: 3.28%
L: 0.35%
5′ 3′
II
T: 0.36%
H: 0.38%
L: 0.36%
T: 3.49%
H: 5.84%
L: 2.92%
T: 4.90%
H: 10.53%
L: 3.56%
T: 5.7%
H: 15.33%
L: 3.37%
5′ 3′
III
T: 0.36%
H: 0.38%
L: 0.36%
T: 3.99%
H: 6.57%
L: 3.37%
T: 5.40%
H: 11.65%
L: 3.92%
T: 6.06%
H: 16.06%
L: 3.63%
5′ 3′
IV
T: 0.43%
H: 0.38%
L: 0.45%
T: 7.77%
H: 15.33%
L: 5.93%
T: 12.38%
H: 25.94%
L: 9.17%
T: 1.43%
H: 5.11%
L: 0.53%
5′ 3′
Bdi
I
T: 0.38%
T: 19.54%
T: 15.09%
T: 6.13%
5′ 3′
Gma
I
T: 0.62%
T: 25.72%
T: 28.09%
T: 1.29%
5′ 3′
Mmu
I
T: 0.00%
H: 0.00%
L: 0.00%
T: 3.42%
H: 7.24%
L: 1.36%
T: 4.80%
H: 10.40%
L: 1.83%
T: 0.68%
H: 1.11%
L: 0.45%
5′ 3′
II
T: 0.00%
H: 0.00%
L: 0.00%
T: 2.74%
H: 5.57%
L: 1.20%
T: 4.30%
H: 9.54%
L: 1.53%
T: 3.13%
H: 6.13%
L: 1.51%
5′ 3′
III
T: 0.00%
H: 0.00%
L: 0.00%
T: 2.74%
H: 5.57%
L: 1.20%
T: 4.40%
H: 9.83%
L: 1.53%
T: 3.42%
H: 6.96%
L: 1.51%
5′ 3′
IV
T: 0.00%
H: 0.00%
L: 0.00%
T: 14.76%
H: 25.63%
L: 8.89%
T: 20.68%
H: 33.82%
L: 13.74%
T: 1.27%
H: 2.23%
L: 0.75%
5′ 3′
Mtr
I
T: 0.24%
T: 8.36%
T: 9.02%
T: 1.31%
5′ 3′
Ppt
I
T: 0.00%
H: 0.00%
L: 0.00%
T: 4.44%
H: 6.45%
L: 2.74%
T: 9.66%
H: 12.70%
L: 7.32%
T: 16.3%
H: 16.13%
L: 16.44%
5′ 3′
Ppe
I
T: 0.00%
T: 0.92%
T: 5.71%
T: 0.00%
5′ 3′
Sly
I
T: 0.00%
H: 0.00%
L: 0.00%
T: 18.87%
H: 13.04%
L: 23.33%
T: 18.37%
H: 22.22%
L: 16.13%
T: 0.00%
H: 0.00%
L: 0.00%
5′ 3′
Stu
I
T: 0.00%
H: 0.00%
L: 0.00%
T: 0.57%
H: 14.29%
L: 0.00%
T: 2.34%
H: 0.00%
L: 2.44%
T: 0.00%
H: 0.00%
L: 0.00%
5′ 3′
Vvi
I
T: 0.00%
H: 0.00%
L: 0.00%
T: 10.87%
H: 20.00%
L: 8.33%
T: 18.18%
H: 13.04%
L: 20.00%
T: 0.00%
H: 0.00%
L: 0.00%
5′ 3′
Zma
I
T: 0.00%
H: 0.00%
L: 0.00%
T: 28.10%
H: 33.33%
L: 26.67%
T: 41.06%
H: 60.61%
L: 35.59%
T: 1.96%
H: 0.00%
L: 2.50%
5′ 3′

Figure S2 Statistical result showing the SPARE (specific parallel amplification of RNA ends) supporting ratios at four processing sites on the microRNA (miRNA) precursors of *Arabidopsis thaliana*. The mature miRNAs are represented by yellow lines on the stem-loop precursor. T: statistical results for all of the processing sites according to the miRBase annotations (release 21). H: statistical results for the processing sites belonging to the high-confidence mature miRNAs registered in miRBase (release 21). L: statistical results for the processing sites belonging to the non-high-confidence mature miRNAs registered in miRBase (release 21). To search for the prominent SPARE signals, the parameter "signal/noise >= 5" was adopted.

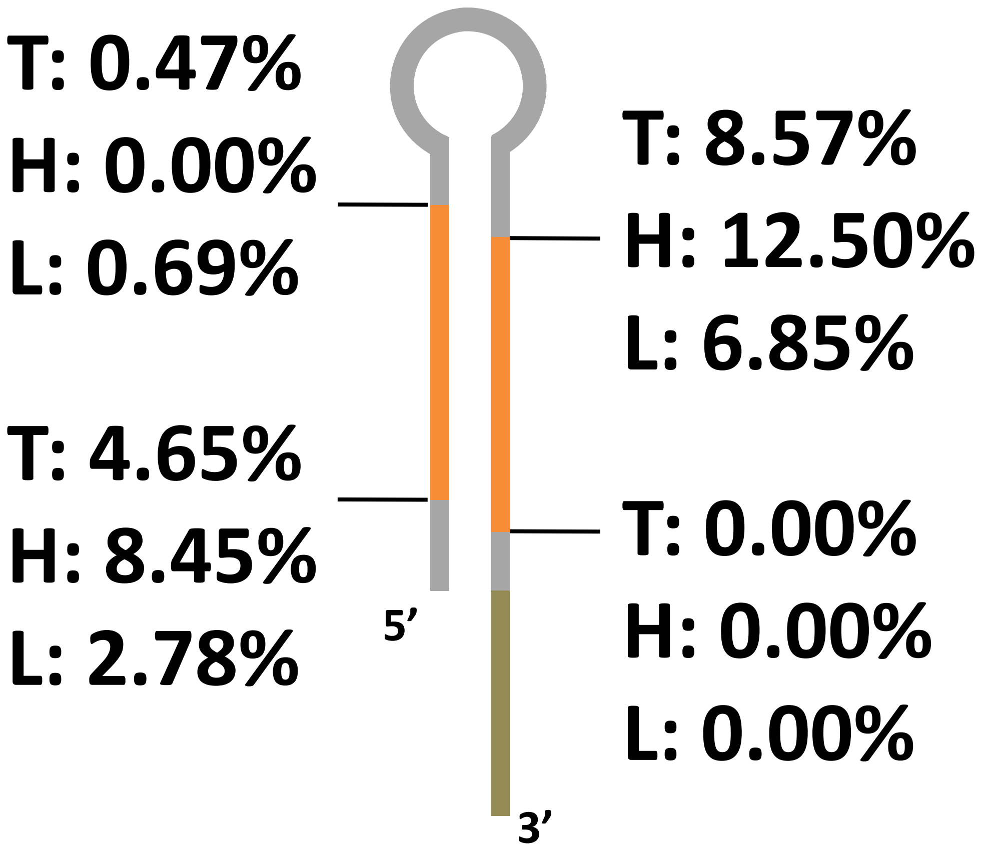
T: 0.47%
H: 0.00%
L: 0.69%
T: 4.65%
H: 8.45%
L: 2.78%
5’
T: 8.57%
H: 12.50%
L: 6.85%
T: 0.00%
H: 0.00%
L: 0.00%
3’

Table S1 Degradome-seq data used in this study.

Species	Dataset ID	Database	Dataset description
Arabidopsis thaliana	GSM278333	GEO (https://www.ncbi.nlm.nih.gov/geo/)	Inflorescence, WT, dT primed, pool-amplified
	GSM278334		
	GSM278335		Inflorescence, WT, random primed, primer extension
	GSM278370		Seedlings, WT, random primed, primer extension
	GSM284751		Inflorescence, WT, control for *ein5*
	GSM284752		Inflorescence, *ein5* mutant
	GSM280226		Inflorescence, WT, control for *xrn4*
	GSM280227		Inflorescence, *xrn4* mutant
	GSM1263708		Inflorescence, WT
	GSM1263709		Inflorescence, *ddm1* mutant
	GSM1263710		Inflorescence, *rdr6* mutant
	GSM1263711		Inflorescence, *ddm1rdr6* double mutant
	GSM2054358		11-day-old seedling, WT
	GSM2054359		Inflorescence, WT
	GSM2253889		WT_rep1_inflorescence
	GSM2253890		*rdr6*_rep1_inflorescence
	GSM2253891		*ago7*_rep1_inflorescence
	GSM2253892		WT_rep2_inflorescence
	GSM2253893		*rdr6*_rep2_inflorescence
	GSM2253894		*ago7*_rep2_inflorescence
	GSM1330569		Young leaf
	GSM1330570		Mature leaf
	GSM1330571		Early senescence leaf biorep 1
	GSM1330572		Late senescence leaf biorep 1
	GSM1330573		Early senescence leaf biorep 2
	GSM1330574		Late senescence leaf biorep 2
Oryza sativa	GSM434596		3-week-old seedling
	GSM455938		
	GSM455939		Young panicle
	GSM1040649		Young panicle at high temperature
Brachypodium distachyon	GSM1173198		12-day-old seedling, control
	GSM1173199		12-day-old seedling, cold treatment
	GSM1173200		12-day-old seedling, control, replicate
	GSM1173201		12-day-old seedling, cold treatment, replicate
	BDI20	Next-Gen Sequence Databases (http://mpss.danforthcenter.org/)	Root
	BDI21		Leaf
	BDI23		Stem
	BDI25		Panicle
Glycine max	GSM848963	GEO	Cotyledon (25-50 mg)
	GSM848964		Seed coat (25-50 mg)
	GSM848965		Cotyledon (100-200 mg)
	GSM848966		Seed coat (100-200 mg)
	GSM848967		Cotyledon (300-400 mg)
	GSM825574		Root, stem, leaf, and inflorescence
	GSM647200		Seed
	GSM1419390		Well-watered leaf
	GSM1419391		Drought-stressed leaf
	GSM1419392		Well-watered leaf, replicate
	GSM1419393		Drought-stressed leaf, replicate
Medicago truncatula	GSM643817		Roots inoculated with Glomus intraradices
	GSM643818		Roots
	GSM769293		Flower
	MEDFL3all	Next-Gen Sequence Databases	
Physcomitrella patens	GSM410805	GEO	10-day-old protonemata, WT
Prunus persica	GSM944975		Mixed tissue
	GSM2193137		Fruit
	GSM1202205		Leave
	GSM1202206		Stem
	GSM1202207		Flower
Solanum lycopersicu	GSM553688		Leaf
	GSM553689		Flower
	GSM553690		Green fruit

Solanum tuberosum	GSM1059071		Tubers, modified stem, 20 ºC
	GSM1059072		Tubers, modified stem, 4 ºC
Vitis vinifera	GSM458931		Leaf
Zea mays	GSM1262608		Mature pollen, WT
	GSM1262609		Mature pollen, *mop1* mutant
	GSM1262610		Mixed sizes of fertile anthers: 1.0 and 1.5 mm
	GSM1262611		Mixed sizes of fertile anthers: 2.0 mm, 2.5 mm and 3.0 mm
	GSM1262612		Mixture of fertile 4.0 mm anthers and mature pollen
	SRX300975	SRA (https://www.ncbi.nlm.nih.gov/sra/)	Young ears, Stage I
	SRX300976		Young ears, Stage II
	SRX300977		Young ears, Stage III
	SRX300978		Young ears, Stage IV
Caenorhabditis elegans	GSM1083459	GEO	Whole organ, WT, adult stage
	GSM1083460		
	GSM1083461		
Drosophila melanogaster	GSM2576151		Adult ovary
	SRR2442926	SRA	S2 cell
	SRR2442923		
Homo sapiens (Step 1)	GSM548630	GEO	HeLa cell-1
	GSM548631		HeLa cell-2
	GSM548632		HeLa cell-3
	GSM548633		HeLa cell-4
	GSM548634		HeLa cell-5
	GSM548635		Whole Brain-1
	GSM548636		Whole Brain-2
	GSM548637		Whole Brain-3
	GSM1666218		Embryonic stem cell line H1
Homo sapiens (Step 2_added data)	GSM1145329		HEK293 cell-1
	GSM1145330		HEK293 cell-2
	GSM1145333		K562 cell-1
	GSM1145334		K562 cell-2
Mus musculus (Step 1)	GSM561025		Brain
	GSM561026		Lung
	GSM561027		Liver
	GSM561028		Kidney
	GSM561029		Ovary
	GSM561030		Spleen
Mus musculus (Step 2_added data)	GSM1666216		Cerebellum
	GSM1666217		Testis

Table S2-1 Tissue-specific expression patterns of the microRNAs supported by cerebellum-specific degradome signatures in *Mus musculus* (signal/noise ratio >= 3).

miRNA ID	miRNA sequence	Expression in GSM1666315_cerebellum_6MPN	Expression in GSM1666319_testis_6MPN	Cerebellum/Testis ratio
mmu-miR-540-3p MIMAT0003167	AGGTCAGAGGTCGATCCTGG	7.40	0.00	
mmu-miR-377-3p MIMAT0000741	ATCACACAAAGGCAACTTTTGT	1.50	0.00	
mmu-miR-344d-3p MIMAT0014808	GATATAACCACTGCCAGACTGA	5.82	0.00	
mmu-miR-338-5p MIMAT0004647	AACAATATCCTGGTGCTGAGTG	3.82	0.00	
mmu-miR-3059-5p MIMAT0014811	TTTCCTCTCTGCCCCATAGGGT	3.99	0.00	
mmu-miR-138-2-3p MIMAT0016987	GCTATTTCACGACACCAGGGT	1.08	0.00	
mmu-miR-193a-5p MIMAT0004544	TGGGTCTTTGCGGGCAAGATGA	1.08	0.00	
mmu-miR-3078-5p MIMAT0014864	CAAAGCCTAGACTGCAGCTACCT	1.91	0.00	
mmu-miR-6540-5p MIMAT0025585	CTAAGGCAGGCAGACTTCAGTG	2.83	0.00	
mmu-miR-129-1-3p MIMAT0016994	AAGCCCTTACCCCAAAAAGTAT	127.71	0.00	
mmu-miR-666-5p MIMAT0003737	AGCGGGCACAGCTGTGAGAGCC	1.16	0.00	
mmu-miR-873a-3p MIMAT0017279	GAGACTGACAAGTTCCCGGGA	3.66	0.00	
mmu-miR-384-5p MIMAT0004745	TGTAAACAATTCCTAGGCAATGT	110.42	0.00	
mmu-miR-668-3p MIMAT0003732	TGTCACTCGGCTCGGCCCACTACC	13.30	0.00	
mmu-miR-212-3p MIMAT0000659	TAACAGTCTCCAGTCACGGCCA	5.32	0.00	
mmu-miR-667-3p MIMAT0003734	TGACACCTGCCACCCAGCCCAAG	2.74	0.00	
mmu-miR-488-3p MIMAT0003450	TTGAAAGGCTGTTTCTTGGTC	2.58	0.00	
mmu-miR-219a-2-3p MIMAT0022841	AGAATTGTGGCTGGACATCTGT	1390.87	2.18	**638.01**
mmu-miR-433-3p MIMAT0001420	ATCATGATGGGCTCCTCGGTGT	733.52	4.73	**155.08**
mmu-miR-598-3p MIMAT0004942	TACGTCATCGTCGTCATCGTTA	266.98	1.82	**146.69**
mmu-miR-434-3p MIMAT0001422	TTTGAACCATCACTCGACTCCT	1209.28	12.74	**94.92**
mmu-miR-132-3p MIMAT0000144	TAACAGTCTACAGCCATGGTCG	187.58	2.55	**73.56**
mmu-miR-124-5p MIMAT0004527	CGTGTTCACAGCGGACCTTGAT	47.39	0.73	**64.92**

mmu-miR-495-3p MIMAT0003456	AAACAAACATGGTGCACTTCTT	108.84	2.18	**49.93**
mmu-miR-370-3p MIMAT0001095	GCCTGCTGGGGTGGAACCTGGT	188.33	4.37	**43.10**
mmu-miR-484 MIMAT0003127	TCAGGCTCAGTCCCCTCCCGAT	294.17	7.64	**38.50**
mmu-miR-181d-5p MIMAT0004324	AACATTCATTGTTGTCGGTGGGT	134.78	4.00	**33.70**
mmu-miR-381-3p MIMAT0000746	TATACAAGGGCAAGCTCTCTGT	3253.02	104.07	**31.26**
mmu-miR-329-5p MIMAT0017032	AGAGGTTTTCTGGGTCTCTGTT	32.18	1.09	**29.52**
mmu-miR-376b-3p MIMAT0001092	ATCATAGAGGAACATCCACTT	41.57	1.46	**28.47**
mmu-miR-335-5p MIMAT0000766	TCAAGAGCAATAACGAAAAATGT	99.94	3.64	**27.46**
mmu-miR-129-2-3p MIMAT0000544	AAGCCCTTACCCCAAAAAGCAT	406.09	17.47	**23.24**
mmu-miR-369-3p MIMAT0003186	AATAATACATGGTTGATCTTT	84.98	4.37	**19.45**
mmu-miR-412-5p MIMAT0017173	TGGTCGACCAGCTGGAAAGTAAT	6.98	0.36	**19.39**
mmu-miR-1198-5p MIMAT0005859	TATGTGTTCCTGGCTGGCTTGG	35.84	2.18	**16.44**
mmu-miR-30a-5p MIMAT0000128	TGTAAACATCCTCGACTGGAAG	3496.05	237.62	**14.71**
mmu-miR-337-5p MIMAT0004644	CGGCGTCATGCAGGAGTTGATT	136.86	9.46	**14.47**
mmu-miR-221-3p MIMAT0000669	AGCTACATTGTCTGCTGGGTTTC	114.91	8.01	**14.35**
mmu-miR-125a-5p MIMAT0000135	TCCCTGAGACCCTTTAACCTGTGA	577.45	42.58	**13.56**
mmu-miR-154-3p MIMAT0004537	AATCATACACGGTTGACCTATT	4.82	0.36	**13.39**
mmu-miR-139-5p MIMAT0000656	TCTACAGTGCACGTGTCTCCAG	12.56	1.09	**11.52**
mmu-miR-30e-5p MIMAT0000248	TGTAAACATCCTTGACTGGAAG	723.79	75.33	**9.61**
mmu-miR-92b-3p MIMAT0004899	TATTGCACTCGTCCCGGCCTCC	96.28	10.55	**9.13**
mmu-miR-181a-5p MIMAT0000210	AACATTCAACGCTGTCGGTGAGT	2774.68	309.68	**8.96**
mmu-miR-135b-5p MIMAT0000612	TATGGCTTTTCATTCCTATGTGA	6.24	0.73	**8.55**
mmu-miR-150-5p MIMAT0000160	TCTCCCAACCCTTGTACCAGTG	27.02	3.28	**8.24**
mmu-miR-664-3p MIMAT0012774	TATTCATTTACTCCCCAGCCTA	2.33	0.36	**6.47**
mmu-miR-187-3p MIMAT0000216	TCGTGTCTTGTGTTGCAGCCGG	2.00	0.36	**5.56**
mmu-miR-181c-3p MIMAT0017068	ACCATCGACCGTTGAGTGGACC	15.22	3.28	**4.64**
mmu-miR-299a-5p MIMAT0000377	TGGTTTACCGTCCCACATACAT	4.32	1.09	**3.96**

mmu-miR-125b-2-3p MIMAT0004529	ACAAGTCAGGTTCTTGGGACCT	7.57	2.18	**3.47**
mmu-miR-107-3p MIMAT0000647	AGCAGCATTGTACAGGGCTATCA	167.37	53.13	**3.15**
mmu-miR-3068-3p MIMAT0014843	GGTGAATTGCAGTACTCCAACA	7.07	2.55	**2.77**
mmu-miR-28a-5p MIMAT0000653	AAGGAGCTCACAGTCTATTGAG	15.96	6.55	**2.44**
mmu-miR-151-3p MIMAT0000161	CTAGACTGAGGCTCCTTGAGG	61.69	25.47	**2.42**
mmu-miR-3081-3p MIMAT0014871	TTGCGCTCCGATCTCTGAGCTGG	1.58	0.73	**2.16**
mmu-miR-125b-1-3p MIMAT0004669	ACGGGTTAGGCTCTTGGGAGCT	10.89	5.09	**2.14**
mmu-miR-146a-5p MIMAT0000158	TGAGAACTGAATTCCATGGGTT	143.76	70.96	**2.03**
mmu-miR-203-3p MIMAT0000236	GTGAAATGTTTAGGACCACTAG	26.27	292.21	**0.09**
mmu-miR-466e-3p MIMAT0004880	TATACATACACGCACACATAAGA	0.33	1.82	**0.18**
mmu-miR-466a-3p MIMAT0002107	TATACATACACGCACACATAAGA	0.33	1.82	**0.18**
mmu-miR-302d-3p MIMAT0003377	TAAGTGCTTCCATGTTTGAGTGT	2.74	13.10	**0.21**
mmu-miR-27a-3p MIMAT0000537	TTCACAGTGGCTAAGTTCCGC	7.82	19.29	**0.41**

Table S2-2 Tissue-specific expression patterns of the microRNAs supported by cerebellum-specific degradome signatures in *Mus musculus* (signal/noise ratio >= 10).

**miRNA ID**	**miRNA sequence**	**Expression in GSM1666315_cerebellum_6MPN**	**Expression in GSM1666319_testis_6MPN**	**Cerebellum/Testis ratio**
mmu-miR-540-3p MIMAT0003167	AGGTCAGAGGTCGATCCTGG	7.40	0.00	
mmu-miR-377-3p MIMAT0000741	ATCACACAAAGGCAACTTTTGT	1.50	0.00	
mmu-miR-3057-5p MIMAT0014822	ATTGGAGCTGAGATTCTGCGGGAT	1.58	0.00	
mmu-miR-666-5p MIMAT0003737	AGCGGGCACAGCTGTGAGAGCC	1.16	0.00	
mmu-miR-338-5p MIMAT0004647	AACAATATCCTGGTGCTGAGTG	3.82	0.00	
mmu-miR-135a-2-3p MIMAT0017064	TGTAGGGATGGAAGCCATGAA	4.07	0.00	
mmu-miR-3059-5p MIMAT0014811	TTTCCTCTCTGCCCCATAGGGT	3.99	0.00	

mmu-miR-873a-3p MIMAT0017279	GAGACTGACAAGTTCCCGGGA	3.66	0.00	
mmu-miR-384-5p MIMAT0004745	TGTAAACAATTCCTAGGCAATGT	110.42	0.00	
mmu-miR-212-3p MIMAT0000659	TAACAGTCTCCAGTCACGGCCA	5.32	0.00	
mmu-miR-667-3p MIMAT0003734	TGACACCTGCCACCCAGCCCAAG	2.74	0.00	
mmu-miR-138-2-3p MIMAT0016987	GCTATTTCACGACACCAGGGT	1.08	0.00	
mmu-miR-193a-5p MIMAT0004544	TGGGTCTTTGCGGGCAAGATGA	1.08	0.00	
mmu-miR-488-3p MIMAT0003450	TTGAAAGGCTGTTTCTTGGTC	2.58	0.00	
mmu-miR-3078-5p MIMAT0014864	CAAAGCCTAGACTGCAGCTACCT	1.91	0.00	
mmu-miR-6540-5p MIMAT0025585	CTAAGGCAGGCAGACTTCAGTG	2.83	0.00	
mmu-miR-219a-2-3p MIMAT0022841	AGAATTGTGGCTGGACATCTGT	1390.87	2.18	**638.01**
mmu-miR-598-3p MIMAT0004942	TACGTCATCGTCGTCATCGTTA	266.98	1.82	**146.69**
mmu-miR-132-3p MIMAT0000144	TAACAGTCTACAGCCATGGTCG	187.58	2.55	**73.56**
mmu-miR-124-5p MIMAT0004527	CGTGTTCACAGCGGACCTTGAT	47.39	0.73	**64.92**
mmu-miR-495-3p MIMAT0003456	AAACAAACATGGTGCACTTCTT	108.84	2.18	**49.93**
mmu-miR-370-3p MIMAT0001095	GCCTGCTGGGGTGGAACCTGGT	188.33	4.37	**43.10**
mmu-miR-222-3p MIMAT0000670	AGCTACATCTGGCTACTGGGT	77.16	1.82	**42.40**
mmu-miR-484 MIMAT0003127	TCAGGCTCAGTCCCCTCCCGAT	294.17	7.64	**38.50**
mmu-miR-181d-5p MIMAT0004324	AACATTCATTGTTGTCGGTGGGT	134.78	4.00	**33.70**
mmu-miR-381-3p MIMAT0000746	TATACAAGGGCAAGCTCTCTGT	3253.02	104.07	**31.26**
mmu-miR-329-5p MIMAT0017032	AGAGGTTTTCTGGGTCTCTGTT	32.18	1.09	**29.52**
mmu-miR-376b-3p MIMAT0001092	ATCATAGAGGAACATCCACTT	41.57	1.46	**28.47**
mmu-miR-335-5p MIMAT0000766	TCAAGAGCAATAACGAAAAATGT	99.94	3.64	**27.46**
mmu-miR-129-2-3p MIMAT0000544	AAGCCCTTACCCCAAAAAGCAT	406.09	17.47	**23.24**
mmu-miR-369-3p MIMAT0003186	AATAATACATGGTTGATCTTT	84.98	4.37	**19.45**
mmu-miR-1198-5p MIMAT0005859	TATGTGTTCCTGGCTGGCTTGG	35.84	2.18	**16.44**

mmu-miR-337-5p MIMAT0004644	CGGCGTCATGCAGGAGTTGATT	136.86	9.46	**14.47**
mmu-miR-221-3p MIMAT0000669	AGCTACATTGTCTGCTGGGTTTC	114.91	8.01	**14.35**
mmu-miR-154-3p MIMAT0004537	AATCATACACGGTTGACCTATT	4.82	0.36	**13.39**
mmu-miR-181a-5p MIMAT0000210	AACATTCAACGCTGTCGGTGAGT	2774.68	309.68	**8.96**
mmu-miR-135b-5p MIMAT0000612	TATGGCTTTTCATTCCTATGTGA	6.24	0.73	**8.55**
mmu-miR-664-3p MIMAT0012774	TATTCATTTACTCCCCAGCCTA	2.33	0.36	**6.47**
mmu-miR-30c-5p MIMAT0000514	TGTAAACATCCTACACTCTCAGC	1280.62	211.42	**6.06**
mmu-miR-299a-5p MIMAT0000377	TGGTTTACCGTCCCACATACAT	4.32	1.09	**3.96**
mmu-miR-331-3p MIMAT0000571	GCCCCTGGGCCTATCCTAGAA	1.33	0.36	**3.69**
mmu-miR-125b-2-3p MIMAT0004529	ACAAGTCAGGTTCTTGGGACCT	7.57	2.18	**3.47**
mmu-miR-3068-3p MIMAT0014843	GGTGAATTGCAGTACTCCAACA	7.07	2.55	**2.77**
mmu-miR-1839-5p MIMAT0009456	AAGGTAGATAGAACAGGTCTTG	184.17	69.87	**2.64**
mmu-miR-28a-5p MIMAT0000653	AAGGAGCTCACAGTCTATTGAG	15.96	6.55	**2.44**
mmu-miR-151-3p MIMAT0000161	CTAGACTGAGGCTCCTTGAGG	61.69	25.47	**2.42**
mmu-miR-3081-3p MIMAT0014871	TTGCGCTCCGATCTCTGAGCTGG	1.58	0.73	**2.16**
mmu-miR-449a-3p MIMAT0017180	CAGCTAACATGCGACTGCTCTC	0.00	2.91	**0.00**
mmu-miR-743b-3p MIMAT0004840	GAAAGACATCATGCTGAATAGA	1.50	537.11	**0.00**
mmu-miR-203-3p MIMAT0000236	GTGAAATGTTTAGGACCACTAG	26.27	292.21	**0.09**
mmu-miR-302d-3p MIMAT0003377	TAAGTGCTTCCATGTTTGAGTGT	2.74	13.10	**0.21**
mmu-miR-27a-3p MIMAT0000537	TTCACAGTGGCTAAGTTCCGC	7.82	19.29	**0.41**
mmu-miR-298-5p MIMAT0000376	GGCAGAGGAGGGCTGTTCTTCCC	6.15	14.19	**0.43**

Table S2-3 Tissue-specific expression patterns of the microRNAs supported by testis-specific degradome signatures in *Mus musculus* (signal/noise ratio >= 3).

miRNA ID	miRNA sequence	Expression in GSM1666315_cerebellum_6MPN	Expression in GSM1666319_testis_6MPN	Testis/Cerebellum ratio
mmu-miR-463-5p MIMAT0002104	TACCTAATTTGTTGTCCATCAT	0.00	50.58	
mmu-miR-201-3p MIMAT0017001	TGAACAGTGCCTTTCTGTGTAGG	0.00	2.91	
mmu-miR-7214-5p MIMAT0028396	TGTTTTCTGGGTTGGAATGAGAA	0.00	8.01	
mmu-miR-471-3p MIMAT0017195	TGAAAGGTGCCATACTATGTAT	0.33	518.19	**1570.27**
mmu-miR-880-3p MIMAT0004844	TACTCCATCCTCTCTGAGTAGA	0.58	443.95	**765.43**
mmu-miR-18a-5p MIMAT0000528	TAAGGTGCATCTAGTGCAGATAG	0.17	16.38	**96.35**
mmu-miR-20a-5p MIMAT0000529	TAAAGTGCTTATAGTGCAGGTAG	78.82	1007.99	**12.79**
mmu-miR-466p-3p MIMAT0014892	ATACATACACGCACACATAAGA	0.58	4.37	**7.53**
mmu-miR-466c-3p MIMAT0004878	ATACATACACGCACACATAAGA	0.58	4.37	**7.53**
mmu-miR-429-3p MIMAT0001537	TAATACTGTCTGGTAATGCCGT	2.74	16.74	**6.11**
mmu-miR-669b-5p MIMAT0003476	AGTTTTGTGTGCATGTGCATGT	0.83	3.64	**4.39**
mmu-miR-25-3p MIMAT0000652	CATTGCACTTGTCTCGGTCTGA	97.70	419.57	**4.29**
mmu-miR-1981-5p MIMAT0009458	GTAAAGGCTGGGCTTAGACGTGGC	2.58	9.10	**3.53**
mmu-miR-148b-3p MIMAT0000580	TCAGTGCATCACAGAACTTTGT	136.03	295.48	**2.17**
mmu-miR-125a-3p MIMAT0004528	ACAGGTGAGGTTCTTGGGAGCC	1.00	0.00	**0.00**
mmu-miR-129-5p MIMAT0000209	CTTTTTGCGGTCTGGGCTTGC	521.41	23.65	**0.05**
mmu-miR-185-5p MIMAT0000214	TGGAGAGAAAGGCAGTTCCTGA	742.25	92.79	**0.13**
mmu-miR-138-5p MIMAT0000150	AGCTGGTGTTGTGAATCAGGCCG	36.25	6.55	**0.18**
mmu-miR-30b-3p MIMAT0004524	CTGGGATGTGGATGTTTACGTC	3.82	1.09	**0.29**
mmu-miR-31-5p MIMAT0000538	AGGCAAGATGCTGGCATAGCTG	5.24	2.55	**0.49**

Table S2-4 Tissue-specific expression patterns of the microRNAs supported by testis-specific degradome signatures in *Mus musculus* (signal/noise ratio >= 10).

**miRNA ID**	**miRNA sequence**	**Expression in GSM1666315_cerebellum_6MPN**	**Expression in GSM1666319_testis_6MPN**	**Testis/Cerebellum ratio**
mmu-miR-463-5p MIMAT0002104	TACCTAATTTGTTGTCCATCAT	0.00	50.58	
mmu-miR-3470b MIMAT0015641	TCACTCTGTAGACCAGGCTGG	0.00	4.37	
mmu-miR-742-3p MIMAT0004237	GAAAGCCACCATGCTGGGTAAA	0.00	2.55	
mmu-miR-201-3p MIMAT0017001	TGAACAGTGCCTTTCTGTGTAGG	0.00	2.91	
mmu-miR-202-3p MIMAT0000235	AGAGGTATAGCGCATGGGAAGA	0.00	10.92	
mmu-miR-471-3p MIMAT0017195	TGAAAGGTGCCATACTATGTAT	0.33	518.19	**1570.27**
mmu-miR-743a-3p MIMAT0004238	GAAAGACACCAAGCTGAGTAGA	1.00	305.31	**305.31**
mmu-miR-18a-5p MIMAT0000528	TAAGGTGCATCTAGTGCAGATAG	0.17	16.38	**96.35**
mmu-miR-34c-3p MIMAT0004580	AATCACTAACCACACAGCCAGG	3.33	133.91	**40.21**
mmu-miR-199b-3p MIMAT0004667	ACAGTAGTCTGCACATTGGTTA	24.61	271.47	**11.03**
mmu-miR-199a-3p MIMAT0000230	ACAGTAGTCTGCACATTGGTTA	24.61	271.47	**11.03**
mmu-miR-17-5p MIMAT0000649	CAAAGTGCTTACAGTGCAGGTAG	15.88	150.65	**9.49**
mmu-miR-92a-3p MIMAT0000539	TATTGCACTTGTCCCGGCCTG	3.82	24.38	**6.38**
mmu-miR-669b-5p MIMAT0003476	AGTTTTGTGTGCATGTGCATGT	0.83	3.64	**4.39**
mmu-miR-1981-5p MIMAT0009458	GTAAAGGCTGGGCTTAGACGTGGC	2.58	9.10	**3.53**
mmu-miR-148b-3p MIMAT0000580	TCAGTGCATCACAGAACTTTGT	136.03	295.48	**2.17**
mmu-miR-9-5p MIMAT0000142	TCTTTGGTTATCTAGCTGTATGA	4425.21	31.30	**0.01**
mmu-miR-382-5p MIMAT0000747	GAAGTTGTTCGTGGTGGATTCG	218.76	2.55	**0.01**
mmu-miR-129-5p MIMAT0000209	CTTTTTGCGGTCTGGGCTTGC	521.41	23.65	**0.05**
mmu-miR-185-5p MIMAT0000214	TGGAGAGAAAGGCAGTTCCTGA	742.25	92.79	**0.13**
mmu-miR-138-5p MIMAT0000150	AGCTGGTGTTGTGAATCAGGCCG	36.25	6.55	**0.18**
mmu-miR-29a-3p MIMAT0000535	TAGCACCATCTGAAATCGGTTA	1439.01	310.77	**0.22**
mmu-miR-31-5p MIMAT0000538	AGGCAAGATGCTGGCATAGCTG	5.24	2.55	**0.49**

Table S3-1 Tissue-specific expression patterns of the microRNAs supported by H1 cell-specific degradome signatures in *Homo sapiens* (signal/noise ratio >= 3).

miRNA ID	miRNA sequence	Expression in GSM494811_K562	Expression in GSM1666320_H1	H1/K562 ratio
hsa-miR-1912 MIMAT0007887	TACCCAGAGCATGCAGTGTGAA	0.00	1.40	
hsa-miR-518b MIMAT0002844	CAAAGCGCTCCCCTTTAGAGGT	0.00	38.76	
hsa-miR-20b-5p MIMAT0001413	CAAAGTGCTCATAGTGCAGGTAG	0.00	2118.69	
hsa-miR-181a-3p MIMAT0000270	ACCATCGACCGTTGATTGTACC	0.00	1.26	
hsa-miR-455-3p MIMAT0004784	GCAGTCCATGGGCATATACAC	0.00	8.95	
hsa-miR-367-3p MIMAT0000719	AATTGCACTTTAGCAATGGTGA	0.00	68.00	
hsa-miR-512-3p MIMAT0002823	AAGTGCTGTCATAGCTGAGGTC	0.00	221.08	
hsa-miR-519a-3p MIMAT0002869	AAAGTGCATCCTTTTAGAGTGT	0.00	8.82	
hsa-miR-187-3p MIMAT0000262	TCGTGTCTTGTGTTGCAGCCGG	0.00	9.37	
hsa-miR-518e-3p MIMAT0002861	AAAGCGCTTCCCTTCAGAGTG	0.00	6.44	
hsa-miR-519c-3p MIMAT0002832	AAAGTGCATCTTTTTAGAGGAT	0.00	2.66	
hsa-miR-302d-3p MIMAT0000718	TAAGTGCTTCCATGTTTGAGTGT	0.00	29494.51	
hsa-miR-429 MIMAT0001536	TAATACTGTCTGGTAAAACCGT	0.00	120.47	
hsa-miR-302c-3p MIMAT0000717	TAAGTGCTTCCATGTTTCAGTGG	0.00	1248.10	
hsa-miR-30a-3p MIMAT0000088	CTTTCAGTCGGATGTTTGCAGC	0.00	2.38	
hsa-miR-520b MIMAT0002843	AAAGTGCTTCCTTTTAGAGGG	0.00	1.96	
hsa-miR-302b-3p MIMAT0000715	TAAGTGCTTCCATGTTTTAGTAG	0.00	48849.32	
hsa-miR-520c-3p MIMAT0002846	AAAGTGCTTCCTTTTAGAGGGT	0.00	35.96	
hsa-miR-195-5p MIMAT0000461	TAGCAGCACAGAAATATTGGC	0.00	1.26	
hsa-miR-520g-3p MIMAT0002858	ACAAAGTGCTTCCCTTTAGAGTGT	0.00	36.94	
hsa-miR-372-3p MIMAT0000724	AAAGTGCTGCGACATTTGAGCGT	0.00	13490.13	

hsa-miR-155-5p MIMAT0000646	TTAATGCTAATCGTGATAGGGGT	0.00	6.02	
hsa-miR-363-3p MIMAT0000707	AATTGCACGGTATCCATCTGTA	0.56	2323.26	**4148.68**
hsa-miR-517a-3p MIMAT0002852	ATCGTGCATCCCTTTAGAGTGT	0.19	238.01	**1252.68**
hsa-miR-517b-3p MIMAT0002857	ATCGTGCATCCCTTTAGAGTGT	0.19	238.01	**1252.68**
hsa-miR-200c-3p MIMAT0000617	TAATACTGCCGGGTAATGATGGA	1.31	717.66	**547.83**
hsa-miR-371a-5p MIMAT0004687	ACTCAAACTGTGGGGGCACT	0.19	82.55	**434.47**
hsa-miR-200b-3p MIMAT0000318	TAATACTGCCTGGTAATGATGA	0.19	36.80	**193.68**
hsa-miR-130a-3p MIMAT0000425	CAGTGCAATGTTAAAAGGGCAT	0.56	37.22	**66.46**
hsa-miR-708-5p MIMAT0004926	AAGGAGCTTACAATCTAGCTGGG	0.37	13.01	**35.16**
hsa-miR-1323 MIMAT0005795	TCAAAACTGAGGGGCATTTTCT	1.31	38.90	**29.69**
hsa-miR-598-3p MIMAT0003266	TACGTCATCGTTGTCATCGTCA	0.56	11.19	**19.98**
hsa-miR-222-3p MIMAT0000279	AGCTACATCTGGCTACTGGGT	1.50	17.07	**11.38**
hsa-miR-653-3p MIMAT0026625	TTCACTGGAGTTTGTTTCAATA	0.19	2.10	**11.05**
hsa-miR-151a-3p MIMAT0000757	CTAGACTGAAGCTCCTTGAGG	29.53	290.20	**9.83**
hsa-miR-92b-3p MIMAT0003218	TATTGCACTCGTCCCGGCCTCC	100.56	912.71	**9.08**
hsa-miR-184 MIMAT0000454	TGGACGGAGAACTGATAAGGGT	0.37	2.52	**6.81**
hsa-miR-548aq-3p MIMAT0022264	CAAAAACTGCAATTACTTTTGC	0.19	1.12	**5.89**
hsa-miR-361-5p MIMAT0000703	TTATCAGAATCTCCAGGGGTAC	6.36	37.36	**5.87**
hsa-miR-379-5p MIMAT0000733	TGGTAGACTATGGAACGTAGG	1.87	9.09	**4.86**
hsa-miR-181b-5p MIMAT0000257	AACATTCATTGCTGTCGGTGGGT	2.24	8.82	**3.94**
hsa-miR-548o-3p MIMAT0005919	CCAAAACTGCAGTTACTTTTGC	3.74	13.85	**3.70**
hsa-miR-129-2-3p MIMAT0004605	AAGCCCTTACCCCAAAAAGCAT	2.99	9.93	**3.32**
hsa-miR-9-5p MIMAT0000441	TCTTTGGTTATCTAGCTGTATGA	18.88	49.11	**2.60**
hsa-miR-1255a MIMAT0005906	AGGATGAGCAAAGAAAGTAGATT	34.21	0.00	**0.00**
hsa-miR-4435 MIMAT0018951	ATGGCCAGAGCTCACACAGAGG	2.06	0.14	**0.07**

hsa-miR-664a-3p MIMAT0005949	TATTCATTTATCCCCAGCCTACA	3.36	0.28	**0.08**
hsa-miR-107 MIMAT0000104	AGCAGCATTGTACAGGGCTATCA	513.09	66.88	**0.13**
hsa-miR-365a-5p MIMAT0009199	AGGGACTTTTGGGGGCAGATGTG	8.97	2.10	**0.23**
hsa-miR-628-5p MIMAT0004809	ATGCTGACATATTTACTAGAGG	2.24	0.56	**0.25**
hsa-miR-941 MIMAT0004984	CACCCGGCTGTGTGCACATGTGC	56.26	24.35	**0.43**

Table S3-2 Tissue-specific expression patterns of the microRNAs supported by H1 cell-specific degradome signatures in *Homo sapiens* (signal/noise ratio >= 10).

miRNA ID	miRNA sequence	Expression in GSM494811_K562	Expression in GSM1666320_H1	H1/K562 ratio
hsa-miR-1912 MIMAT0007887	TACCCAGAGCATGCAGTGTGAA	0.00	1.40	
hsa-miR-518b MIMAT0002844	CAAAGCGCTCCCCTTTAGAGGT	0.00	38.76	
hsa-miR-302d-3p MIMAT0000718	TAAGTGCTTCCATGTTTGAGTGT	0.00	29494.51	
hsa-miR-20b-5p MIMAT0001413	CAAAGTGCTCATAGTGCAGGTAG	0.00	2118.69	
hsa-miR-429 MIMAT0001536	TAATACTGTCTGGTAAAACCGT	0.00	120.47	
hsa-miR-302c-3p MIMAT0000717	TAAGTGCTTCCATGTTTCAGTGG	0.00	1248.10	
hsa-miR-181a-3p MIMAT0000270	ACCATCGACCGTTGATTGTACC	0.00	1.26	
hsa-miR-455-3p MIMAT0004784	GCAGTCCATGGGCATATACAC	0.00	8.95	
hsa-miR-30a-3p MIMAT0000088	CTTTCAGTCGGATGTTTGCAGC	0.00	2.38	
hsa-miR-367-3p MIMAT0000719	AATTGCACTTTAGCAATGGTGA	0.00	68.00	
hsa-miR-520b MIMAT0002843	AAAGTGCTTCCTTTTAGAGGG	0.00	1.96	
hsa-miR-512-3p MIMAT0002823	AAGTGCTGTCATAGCTGAGGTC	0.00	221.08	
hsa-miR-302b-3p MIMAT0000715	TAAGTGCTTCCATGTTTTAGTAG	0.00	48849.32	
hsa-miR-519a-3p MIMAT0002869	AAAGTGCATCCTTTTAGAGTGT	0.00	8.82	
hsa-miR-520c-3p MIMAT0002846	AAAGTGCTTCCTTTTAGAGGGT	0.00	35.96	

hsa-miR-195-5p MIMAT0000461	TAGCAGCACAGAAATATTGGC	0.00	1.26		
hsa-miR-518e-3p MIMAT0002861	AAAGCGCTTCCCTTCAGAGTG	0.00	6.44		
hsa-miR-520g-3p MIMAT0002858	ACAAAGTGCTTCCCTTTAGAGTGT	0.00	36.94		
hsa-miR-519c-3p MIMAT0002832	AAAGTGCATCTTTTTAGAGGAT	0.00	2.66		
hsa-miR-363-3p MIMAT0000707	AATTGCACGGTATCCATCTGTA	0.56	2323.26	**4148.68**	
hsa-miR-200c-3p MIMAT0000617	TAATACTGCCGGGTAATGATGGA	1.31	717.66	**547.83**	
hsa-miR-371a-5p MIMAT0004687	ACTCAAACTGTGGGGGCACT	0.19	82.55	**434.47**	
hsa-miR-200b-3p MIMAT0000318	TAATACTGCCTGGTAATGATGA	0.19	36.80	**193.68**	
hsa-miR-148a-3p MIMAT0000243	TCAGTGCACTACAGAACTTTGT	123.18	11045.42	**89.67**	
hsa-miR-130a-3p MIMAT0000425	CAGTGCAATGTTAAAAGGGCAT	0.56	37.22	**66.46**	
hsa-miR-708-5p MIMAT0004926	AAGGAGCTTACAATCTAGCTGGG	0.37	13.01	**35.16**	
hsa-miR-1323 MIMAT0005795	TCAAAACTGAGGGGCATTTTCT	1.31	38.90	**29.69**	
hsa-miR-598-3p MIMAT0003266	TACGTCATCGTTGTCATCGTCA	0.56	11.19	**19.98**	
hsa-miR-653-3p MIMAT0026625	TTCACTGGAGTTTGTTTCAATA	0.19	2.10	**11.05**	
hsa-miR-151a-3p MIMAT0000757	CTAGACTGAAGCTCCTTGAGG	29.53	290.20	**9.83**	
hsa-miR-184 MIMAT0000454	TGGACGGAGAACTGATAAGGGT	0.37	2.52	**6.81**	
hsa-miR-548aq-3p MIMAT0022264	CAAAAACTGCAATTACTTTTGC	0.19	1.12	**5.89**	
hsa-miR-361-5p MIMAT0000703	TTATCAGAATCTCCAGGGGTAC	6.36	37.36	**5.87**	
hsa-miR-379-5p MIMAT0000733	TGGTAGACTATGGAACGTAGG	1.87	9.09	**4.86**	
hsa-miR-181b-5p MIMAT0000257	AACATTCATTGCTGTCGGTGGGT	2.24	8.82	**3.94**	
hsa-miR-548o-3p MIMAT0005919	CCAAAACTGCAGTTACTTTTGC	3.74	13.85	**3.70**	
hsa-miR-129-2-3p MIMAT0004605	AAGCCCTTACCCCAAAAAGCAT	2.99	9.93	**3.32**	
hsa-miR-9-5p MIMAT0000441	TCTTTGGTTATCTAGCTGTATGA	18.88	49.11	**2.60**	
hsa-miR-1255a MIMAT0005906	AGGATGAGCAAAGAAAGTAGATT	34.21	0.00	**0.00**	
hsa-let-7g-5p MIMAT0000414	TGAGGTAGTAGTTTGTACAGTT	728.42	31.06	**0.04**	

hsa-miR-4435 MIMAT0018951	ATGGCCAGAGCTCACACAGAGG	2.06	0.14	**0.07**
hsa-miR-664a-3p MIMAT0005949	TATTCATTTATCCCCAGCCTACA	3.36	0.28	**0.08**
hsa-miR-107 MIMAT0000104	AGCAGCATTGTACAGGGCTATCA	513.09	66.88	**0.13**
hsa-miR-365a-5p MIMAT0009199	AGGGACTTTTGGGGGCAGATGTG	8.97	2.10	**0.23**
hsa-miR-628-5p MIMAT0004809	ATGCTGACATATTTACTAGAGG	2.24	0.56	**0.25**
hsa-miR-941 MIMAT0004984	CACCCGGCTGTGTGCACATGTGC	56.26	24.35	**0.43**

Table S3-3 Tissue-specific expression patterns of the microRNAs supported by K562 cell-specific degradome signatures in *Homo sapiens* (signal/noise ratio >= 3).

miRNA ID	miRNA sequence	Expression in GSM494811_K562	Expression in GSM1666320_H1	K562/H1 ratio
hsa-miR-3179 MIMAT0015056	AGAAGGGGTGAAATTTAAACGT	2.80	0.00	
hsa-miR-1277-3p MIMAT0005933	TACGTAGATATATATGTATTTT	5.61	0.00	
hsa-miR-1255b-5p MIMAT0005945	CGGATGAGCAAAGAAAGTGGTT	17.76	0.00	
hsa-miR-642a-3p MIMAT0020924	AGACACATTTGGAGAGGGAACC	44.49	0.00	
hsa-miR-1268a MIMAT0005922	CGGGCGTGGTGGTGGGGG	6.73	0.00	
hsa-let-7e-5p MIMAT0000066	TGAGGTAGGAGGTTGTATAGTT	2474.43	4.20	**589.15**
hsa-miR-185-5p MIMAT0000455	TGGAGAGAAAGGCAGTTCCTGA	8547.80	100.46	**85.09**
hsa-miR-423-5p MIMAT0004748	TGAGGGGCAGAGAGCGAGACTTT	4872.23	60.87	**80.04**
hsa-miR-766-5p MIMAT0022714	AGGAGGAATTGGTGCTGGTCTT	9.16	0.14	**65.43**
hsa-miR-576-3p MIMAT0004796	AAGATGTGGAAAAATTGGAATC	8.41	0.28	**30.04**
hsa-miR-1246 MIMAT0005898	AATGGATTTTTGGAGCAGG	43.93	2.38	**18.46**
hsa-miR-193b-5p MIMAT0004767	CGGGGTTTTGAGGGCGAGATGA	12.34	0.70	**17.63**
hsa-miR-486-5p MIMAT0002177	TCCTGTACTGAGCTGCCCCGAG	402.62	23.23	**17.33**
hsa-miR-548l MIMAT0005889	AAAAGTATTTGCGGGTTTTGTC	2.06	0.14	**14.71**

hsa-miR-1270 MIMAT0005924	CTGGAGATATGGAAGAGCTGTGT	5.23	0.42	**12.45**
hsa-miR-550a-3p MIMAT0003257	TGTCTTACTCCCTCAGGCACAT	3.36	0.28	**12.00**
hsa-miR-505-5p MIMAT0004776	GGGAGCCAGGAAGTATTGATGT	2.99	0.28	**10.68**
hsa-miR-1304-5p MIMAT0005892	TTTGAGGCTACAGTGAGATGTG	1.12	0.42	**2.67**
hsa-miR-151a-5p MIMAT0004697	TCGAGGAGCTCACAGTCTAGT	31.03	15.25	**2.03**
hsa-miR-4664-3p MIMAT0019738	CTTCCGGTCTGTGAGCCCCGTC	0.00	1.12	**0.00**
hsa-miR-125b-1-3p MIMAT0004592	ACGGGTTAGGCTCTTGGGAGCT	0.00	4.06	**0.00**
hsa-miR-183-5p MIMAT0000261	TATGGCACTGGTAGAATTCACT	17.01	187.63	**0.09**
hsa-miR-143-3p MIMAT0000435	TGAGATGAAGCACTGTAGCTC	2.24	17.49	**0.13**
hsa-miR-27b-3p MIMAT0000419	TTCACAGTGGCTAAGTTCTGC	42.43	326.16	**0.13**
hsa-miR-30d-3p MIMAT0004551	CTTTCAGTCAGATGTTTGCTGC	0.19	1.40	**0.14**
hsa-miR-30c-5p MIMAT0000244	TGTAAACATCCTACACTCTCAGC	8.41	60.87	**0.14**
hsa-miR-99b-3p MIMAT0004678	CAAGCTCGTGTCTGTGGGTCCG	2.06	13.99	**0.15**
hsa-miR-652-3p MIMAT0003322	AATGGCGCCACTAGGGTTGTG	3.36	11.05	**0.30**
hsa-miR-18a-5p MIMAT0000072	TAAGGTGCATCTAGTGCAGATAG	38.88	127.47	**0.31**
hsa-miR-590-3p MIMAT0004801	TAATTTTATGTATAAGCTAGT	1.87	5.88	**0.32**
hsa-miR-221-3p MIMAT0000278	AGCTACATTGTCTGCTGGGTTTC	146.54	400.32	**0.37**

Table S3-4 Tissue-specific expression patterns of the microRNAs supported by K562 cell-specific degradome signatures in *Homo sapiens* (signal/noise ratio >= 10).

miRNA ID	miRNA sequence	Expression in GSM494811_K562	Expression in GSM1666320_H1	K562/H1 ratio
hsa-miR-3179 MIMAT0015056	AGAAGGGGTGAAATTTAAACGT	2.80	0.00	
hsa-miR-1277-3p MIMAT0005933	TACGTAGATATATATGTATTTT	5.61	0.00	
hsa-miR-1255b-5p MIMAT0005945	CGGATGAGCAAAGAAAGTGGTT	17.76	0.00	

hsa-miR-642a-3p MIMAT0020924	AGACACATTTGGAGAGGGAACC	44.49	0.00	
hsa-miR-1268a MIMAT0005922	CGGGCGTGGTGGTGGGGG	6.73	0.00	
hsa-let-7e-5p MIMAT0000066	TGAGGTAGGAGGTTGTATAGTT	2474.43	4.20	**589.15**
hsa-miR-185-5p MIMAT0000455	TGGAGAGAAAGGCAGTTCCTGA	8547.80	100.46	**85.09**
hsa-miR-766-5p MIMAT0022714	AGGAGGAATTGGTGCTGGTCTT	9.16	0.14	**65.43**
hsa-miR-576-3p MIMAT0004796	AAGATGTGGAAAAATTGGAATC	8.41	0.28	**30.04**
hsa-miR-1246 MIMAT0005898	AATGGATTTTTGGAGCAGG	43.93	2.38	**18.46**
hsa-miR-548l MIMAT0005889	AAAAGTATTTGCGGGTTTTGTC	2.06	0.14	**14.71**
hsa-miR-1270 MIMAT0005924	CTGGAGATATGGAAGAGCTGTGT	5.23	0.42	**12.45**
hsa-miR-550a-3p MIMAT0003257	TGTCTTACTCCCTCAGGCACAT	3.36	0.28	**12.00**
hsa-miR-505-5p MIMAT0004776	GGGAGCCAGGAAGTATTGATGT	2.99	0.28	**10.68**
hsa-miR-142-3p MIMAT0000434	TGTAGTGTTTCCTACTTTATGGA	5.23	0.56	**9.34**
hsa-miR-210-3p MIMAT0000267	CTGTGCGTGTGACAGCGGCTGA	70.09	14.55	**4.82**
hsa-miR-1304-5p MIMAT0005892	TTTGAGGCTACAGTGAGATGTG	1.12	0.42	**2.67**
hsa-miR-151a-5p MIMAT0004697	TCGAGGAGCTCACAGTCTAGT	31.03	15.25	**2.03**
hsa-miR-4664-3p MIMAT0019738	CTTCCGGTCTGTGAGCCCCGTC	0.00	1.12	**0.00**
hsa-miR-125b-1-3p MIMAT0004592	ACGGGTTAGGCTCTTGGGAGCT	0.00	4.06	**0.00**
hsa-miR-143-3p MIMAT0000435	TGAGATGAAGCACTGTAGCTC	2.24	17.49	**0.13**
hsa-miR-27b-3p MIMAT0000419	TTCACAGTGGCTAAGTTCTGC	42.43	326.16	**0.13**
hsa-miR-30d-3p MIMAT0004551	CTTTCAGTCAGATGTTTGCTGC	0.19	1.40	**0.14**
hsa-miR-30c-5p MIMAT0000244	TGTAAACATCCTACACTCTCAGC	8.41	60.87	**0.14**
hsa-miR-99b-3p MIMAT0004678	CAAGCTCGTGTCTGTGGGTCCG	2.06	13.99	**0.15**
hsa-miR-362-5p MIMAT0000705	AATCCTTGGAACCTAGGTGTGAGT	3.74	16.09	**0.23**
hsa-miR-652-3p MIMAT0003322	AATGGCGCCACTAGGGTTGTG	3.36	11.05	**0.30**
hsa-miR-221-3p MIMAT0000278	AGCTACATTGTCTGCTGGGTTTC	146.54	400.32	**0.37**
